# **Prediction of coal mine water conduction fracture zone height based on integrated learning model**

**DOI:** 10.1038/s41598-025-13627-7

**Published:** 2025-08-01

**Authors:** Meng Wang, Xufeng Zhang, Xin Li, Zhongzheng Fang, Yafei Wei, Hongyan Qin, Shuai Wang, Tianyu Liu, Yaqiang Zheng

**Affiliations:** 1 College of Mining, Liaoning Technical University, Fuxin, 123000 Liaoning China; 2 Xima Mine of Shenyang Coking Coal Co., Ltd, Liaoyang, 111000 Liaoning China; 3 North China Institute of Science and Technology, Langfang, 065201 Hebei China

**Keywords:** Water-conducting fracture zone, SVR, XGBoost, Integrated learning, Artificial intelligence, Coal, Hydrology

## Abstract

To enhance the accuracy of predicting the height of water-conducting fracture zones (WCFZ) in coal mines, this study proposes a novel stacked ensemble learning model. The model integrates XGBoost and Support Vector Regression (SVR) as base learners, with CatBoost serving as the meta-learner, forming a two-layer architecture. Key geological and mining features—such as mining height, burial depth, working face length, and lithologic proportion coefficient—are used as input variables to better capture the complex influencing factors. Validation using data from the No. 3 coal seam of Husheng Coal Mine demonstrates that the proposed model achieves a predicted WCFZ height of 50.79 m, closely aligning with the measured value and outperforming empirical formulas (61.4 m), standalone SVR (58.14 m), XGBoost (56.62 m), and FLAC3D simulation (55 m). The model also achieves an R² of 0.98 and RMSE of 2.08, indicating excellent predictive performance. This research is the first to introduce stacked ensemble learning for WCFZ height prediction, overcoming the limitations of single-model and simulation-based methods. The proposed approach offers a more accurate and intelligent tool for mine water hazard assessment and represents a significant advancement in applying machine learning to underground geological engineering.

## Introduction

With the advance of the mining of the working face, the balance state of the original rock stress of the overlying rock is damaged, resulting in the overlying rock collapse, fracture, bending and other phenomena. Once the water-conducting fissure zone in the goaf touches the aquifer or reaches the surface, it will likely cause safety accidents such as sand and water collapse. Therefore, studying the development height of a water-conducting fracture zone is of positive significance.

The prediction of the height of the water-conducting fracture zone in China mainly includes empirical formula, theoretical calculation, similar material simulation, numerical simulation, machine learning, neural network, field measurement, etc^1^. Among them, the field measurement method has the highest accuracy but is time-consuming, laborious and costly^2^. The model established by the theoretical calculation method must be more idealized, which deviates greatly from the complex actual geological occurrence conditions^3^. A similar material simulation method has high requirements for the accuracy of material ratio, and it isn’t easy to simulate some complex geological conditions^4^. The accuracy of the numerical simulation method is closely related to the parameters of geological conditions for establishing the model, and it isn’t easy to obtain the parameters accurately^5^. The empirical formula in the “three-down” mining procedure considers a single influencing factor, which cannot reflect the comprehensive effect of multiple influencing factors^6^. Although the neural network method considers various influencing factors, it is easy to generate local optimal solutions. The accuracy is low when the samples are small, and it is easy to over-learn when the samples are large, resulting in the low generalization ability of the model^7^. Machine learning, developed based on statistical learning theory, is based on small-sample learning and global optimization. It has the characteristics of high precision, fast convergence speed, and strong generalization ability, and it can provide positive help in predicting the height of the water-conducting fracture zone^8^.

In recent years, some scholars have used the method of multi-factor superposition analysis to predict the water-conducting fracture zone in complex regions. Xu Jialin et al.^9^ proposed a theoretical method to estimate the height of the water-conducting fracture zone by the location of the key layer of overlying rock. Subsequently, some scholars concluded through summary and induction that the control factors for the development height of the water-conducting fracture zone include coal seam burial depth, working face oblique length, coal seam inclination, etc^10^. The research on the height of the water-conducting fracture zone also gradually transitioned from a single method to a multi-method comprehensive analysis and determination^11^. Yin Shangxian^12^ and Xu Yanchun^13^ et al. collected a large number of measured height data of water-guiding fracture zones, analyzed the development height of water-guiding fracture zones under different overburden conditions, and established regression prediction models of water-guiding fracture zone heights under corresponding conditions to improve the prediction accuracy of the multivariate linear model. Hu Xiaojuan et al.^14^ established a linear multiple regression prediction model that considers factors such as mining thickness and burial depth for prediction. Shi Longqing et al.^15^ established the PCA-GA-Elman model considering the correlation among factors. Still, they ignored the influence of the proportion coefficient of roof overlying rock on the water-conducting fracture zone.

Zhao Zhongming et al.^16^ established an ANN model to optimize the prediction of the height of the water-conducting fracture zone by considering secondary factors such as burial depth and hard rock proportion coefficient. Zhang Heng^17^ conducted a comprehensive comparative analysis of the development height of the water-conducting fracture zone through parallel network electrical dynamic monitoring of overburden rock failure combined with numerical simulation and found that the empirical formula needed to be more applicable in weakly cemented areas. By combining fractal geometry theory with numerical simulation, Chen Kai et al.^18^ revealed the evolution characteristics of the mining-induced fracture network of weakly cemented overburden in the western super-thick coal seam. Wu Jianhong et al.^19^ took the Jurassic coal field of Huanglong as the research area, considered the influence factors of the water-conducting fracture zone and established a prediction model of the height of the water-conducting fracture zone applicable to the Huanglong coal field based on the data drive. Ma Junqian et al.^20^ took the Yili No. 4 mine in Xinjiang as the research background. Through similar simulation experiments, they revealed the law of overburden fracture and fracture development after strata mining. Sun Lihui et al.^21^ analyzed weakly consolidated rock’s physical and mechanical properties using field investigation, theoretical analysis and laboratory measurement. They found that the strong disintegration of weakly consolidated rock was the main reason for the increase in the height of the water-conducting fracture zone. Shi Shouqiao et al.^22^ improved the empirical calculation formula of the height of the water-conducting fracture zone through regression analysis. They improved the prediction accuracy of the height of the water-conducting fracture zone. Zhang Yandong^23^ obtained a guide height calculation formula suitable for the Yusuling Coal mine by synthesizing and comparing the development height of the zone under different methods and revising the existing empirical formula; Based on the measured data, Wang Xu et al.^24^ adopted factor analysis to establish a prediction model of the height of water-conducting fracture zone, such as multiple regression and BP neural network. Xu Zhimin et al.^25^ used various methods to reveal the failure process and evolution characteristics of coal seam mining roofs in Xinjiang. They fitted and modified the existing empirical formulas of water-conductivity fracture zones. Zhang Hongwei et al.^26^ used the improved fruit fly optimization algorithm to optimize parameters and establish a support vector machine model to provide a theoretical basis for predicting water-conducting fracture zones in other mining areas. Zhang Fengda et al.^27^ combined a particle swarm optimization algorithm with a support vector machine to establish a mathematical model of the failure depth of deep coal seam floor. Liu Peng et al.^28^ combined a support vector machine to propose an enhanced CART retrieval algorithm to effectively improve the prediction accuracy of gas emissions in coal faces. The studies of these scholars have provided new ideas for predicting water-conductivity fracture zones.

In recent years, Support Vector Machine (SVM) and Extreme Gradient Boosting (XGBoost) have been widely applied in fields such as geotechnical engineering and underground structure prediction. Especially in dealing with nonlinear and multivariable coupled prediction problems, they have demonstrated high accuracy and robustness. Gharabaghi et al.^29^ successfully estimated the consumption of foaming agents (surfactants) in earth pressure balance shield machines by using statistical and soft computing methods, effectively enhancing the scientificity and efficiency of construction parameter regulation. Zhang et al.^30^ developed two robust hybrid models specifically for predicting tunnel deformation problems in weakly deformed strata, which is of great significance in improving the generalization ability and prediction accuracy of the models. These studies provide strong support for applying SVM and XGBoost to prediction problems under complex geological conditions.

The latest research shows that ensemble learning methods perform particularly well when dealing with highly nonlinear geological parameters and small sample problems. Zhao et al.^31^ proposed an integrated algorithm based on the fusion of Bagging and Boosting in Flow Measurement and Instrumentation to predict the development height of coal seam fracture zones, significantly improving the stability and generalization ability of the model. Furthermore, Wang et al.^32^ constructed an interpretable deep neural network model (XAI-DNN) in Computers and Structures for predicting the risk of water instep in coal mines. This not only improved the prediction performance but also achieved the transparency and interpretability of the model through the SHAP value. It provides theoretical support for the identification of high-risk areas. Abdelrahman Kamal Hamed et al. combined three basic models, namely neural networks, random forests and extreme gradient boosting, with linear regression to predict compressive strength.

In summary, based on the empirical formula to calculate the development height of a water-conducting fracture zone, this method can not give specific calculation formulas for different geological mining areas^33^. There are many characteristics of the development height of a water-conducting fracture zone, and only a single factor is considered. Hence, the prediction results of the empirical formula method need to be more accurate. The numerical simulation and multivariate prediction methods incorporate more features into the model calculation and analyze and predict the data through the machine learning model, which improves the model prediction accuracy compared with the empirical formula method. However, in terms of machine learning for predicting the height of water-conducting fracture zones, most studies have focused on eXtreme Gradient Boosting models and support vector machines. Among them, the core idea of the Support Vector Machine (SVM) model is to find an optimal hyperplane, separate data of different categories, and maximize the interval from the two types of data to the hyperplane. To enhance the generalization ability of the model. The core of eXtreme Gradient Boosting is to gradually improve the model performance by iteratively training multiple weak learners (decision trees) and optimizing the residuals of the prediction results. Hereinafter referred to as SVM and XGBoost models for short. But may need to perform better when dealing with data with strong linear relationships. The XGBoost model does not handle high-dimensional sparse data like other models. To overcome the shortcomings of SVR and XGBoost models, some scholars have carried out algorithm improvement and optimization on the model itself, greatly improving its accuracy^34^. Such optimization methods still need to change the model’s shortcomings. Therefore, in this study, Categorical Boosting is chosen as the meta-model, and XGBoost and SVM are used as the basic models to establish an integrated model. Categorical Boosting has certain advantages in dealing with categorical features and high-dimensional sparse data, and can effectively reduce the risk of overfitting at the same time. Hereinafter referred to as CatBoost. In the integrated model, SVR can capture nonlinear patterns in the data, XGBoost can handle complex feature interactions, and CatBoost can integrate their predictions and learn how to combine them best, making up for some shortcomings in the model itself.In addition to traditional and machine learning-based approaches, recent interdisciplinary studies have enriched the theoretical and technical foundations of predicting the height of water-conducting fracture zones. For instance, Li et al.^35^ investigated slope instability mechanisms in open-pit coal mines with weak interlayers, emphasizing the rock arching effect and crack propagation, which offer theoretical insights into the fracture evolution process in underground coal mining. Similarly, Wang et al.^36^ explored the mechanical behavior of narrow coal pillars under hard roof conditions, shedding light on stress redistribution patterns and surrounding rock deformation, which are crucial factors influencing fracture zone development. These findings highlight the importance of roadway layout and coal pillar design as potential input features for predictive models.Meanwhile, Wang et al.^37^ developed an industrial IoT-based emulsion dispensing and remote monitoring system for coal mine operations, demonstrating the feasibility of real-time data acquisition and environmental sensing, which can provide high-quality inputs for machine learning models in geotechnical prediction.Furthermore, Wang et al.^38^ examined the anchorage failure mechanisms of prestressed rock bolt systems, revealing that anchorage length, interfacial shear stiffness, and axial force significantly affect surrounding rock stability and fracture propagation. These mechanical parameters should be considered in predictive modeling of water-conducting fracture zone height.Beyond coal mining, Armanuos and Elshaarawy^39^ applied explainable boosting models (e.g., XGBoost + SHAP) to predict saltwater intrusion in sloping aquifers, demonstrating strong performance in capturing nonlinear hydrogeological interactions. These findings support the use of similar integrated models in coal mines, where complex subsurface conditions prevail.

In civil engineering, Abdelrahman et al.^40^ used stacked machine learning models to predict concrete compressive strength, confirming the high accuracy and adaptability of ensemble models for multi-feature problems. Likewise, Elshaarawy and Hamed^41^ achieved significant improvements in predicting triangular side orifice discharge coefficients by combining SVR, RF, and XGBoost under an ANN metamodel, demonstrating the robustness of stacked ensemble frameworks in fluid-structure systems. These interdisciplinary advances collectively validate the integration of SVR, XGBoost, and CatBoost into a meta-learner ensemble for predicting the height of water-conducting fracture zones.

## Overview of the study area

According to the Paleozoic stratigraphic map of Inner Mongolia, the research area belongs to the Dalai-Xingan stratigraphic area in the Northern Xinjiang-Xingan stratigraphic region. The strata developed in the region are the Paleozoic Upper Devonian metamorphic rock series, Mesozoic Upper Jurassic Baiyingaolao Formation, Lower Cretaceous Lower Cretaceous Meletu Formation and Damaoguaihe Formation, Quaternary system.

The minefield is near a shallowly buried coal seam, and this area has four layers of mostly and partially minable coal seams. The coal seam numbers are 1–2 + 3 (1–2), 2 − 1 + 2 (2–2), 3 (3 − 1) and 3 − 2 + 3 coal seams from top to bottom. The mine is mainly mining No. 3 coal seam, which is currently being mined. The overall occurrence of the coal seam is stable. Comprehensive mechanized top-caving coal mining technology has been adopted.

**Fig. 1:**
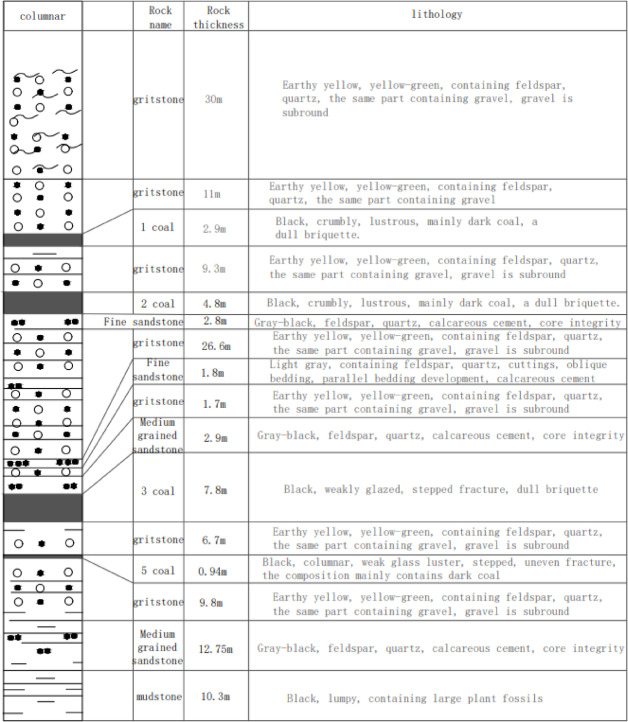
Rock column diagram.

To analyze the height of the water-conducting fracture zone of the mining face and predict the water-conducting fracture zone of the finished working face, the 316 working face that has completed the mining task was measured by using sublevel water injection borehole detection method in the underground uphole. The average mining height of 316 working faces is 7.8 m, the inclination length is 174 m, the strike length is 1093 m, the inclination is 1–3 °, and the strike of the coal seam is 175°-185°. Within the working face, the coal seam structure is simple, the gangue is generally 1–2 layers, and the bedding and joints are developed, which does not influence mining. The coal seam column diagram is shown in Fig. 1.

**Fig. 2:**
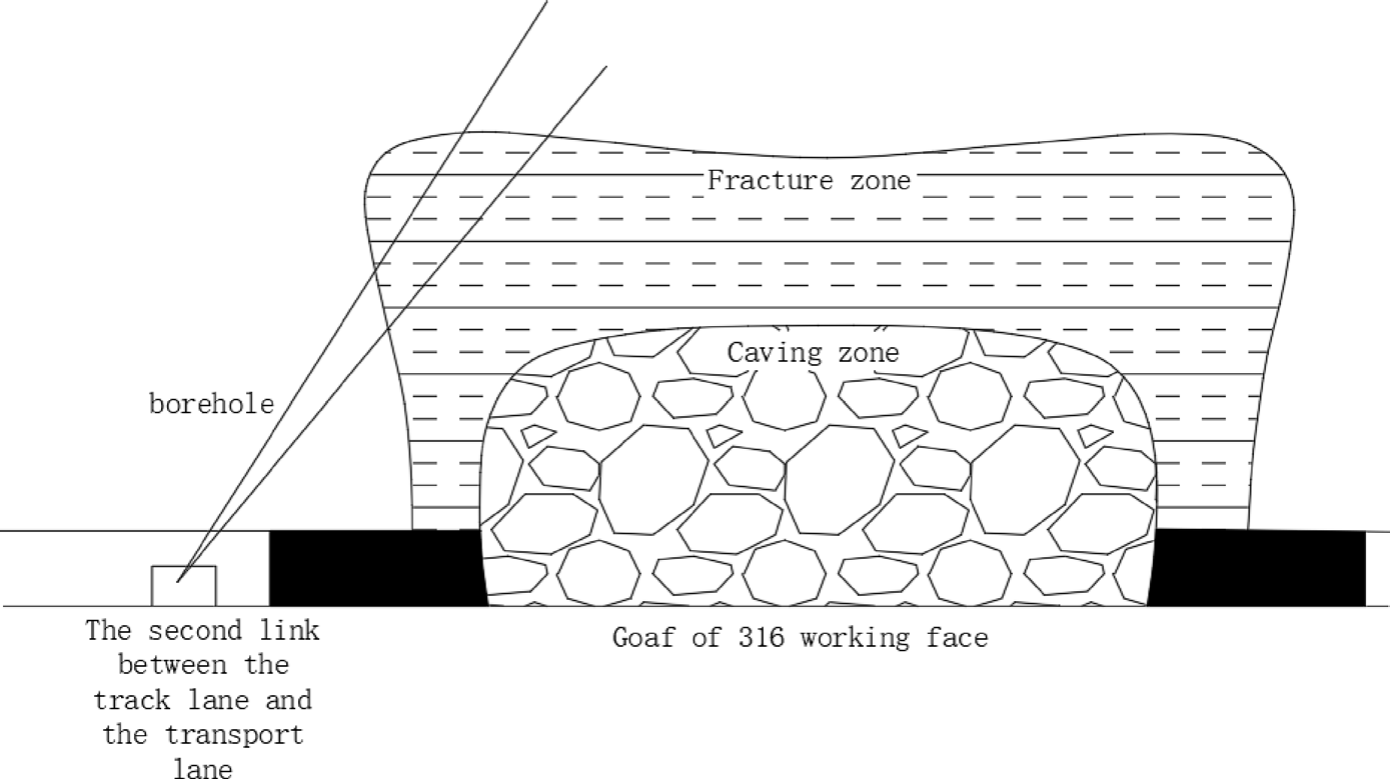
Schematic diagram of the observation method of sub-level water injection borehole.

**Fig. 3:**
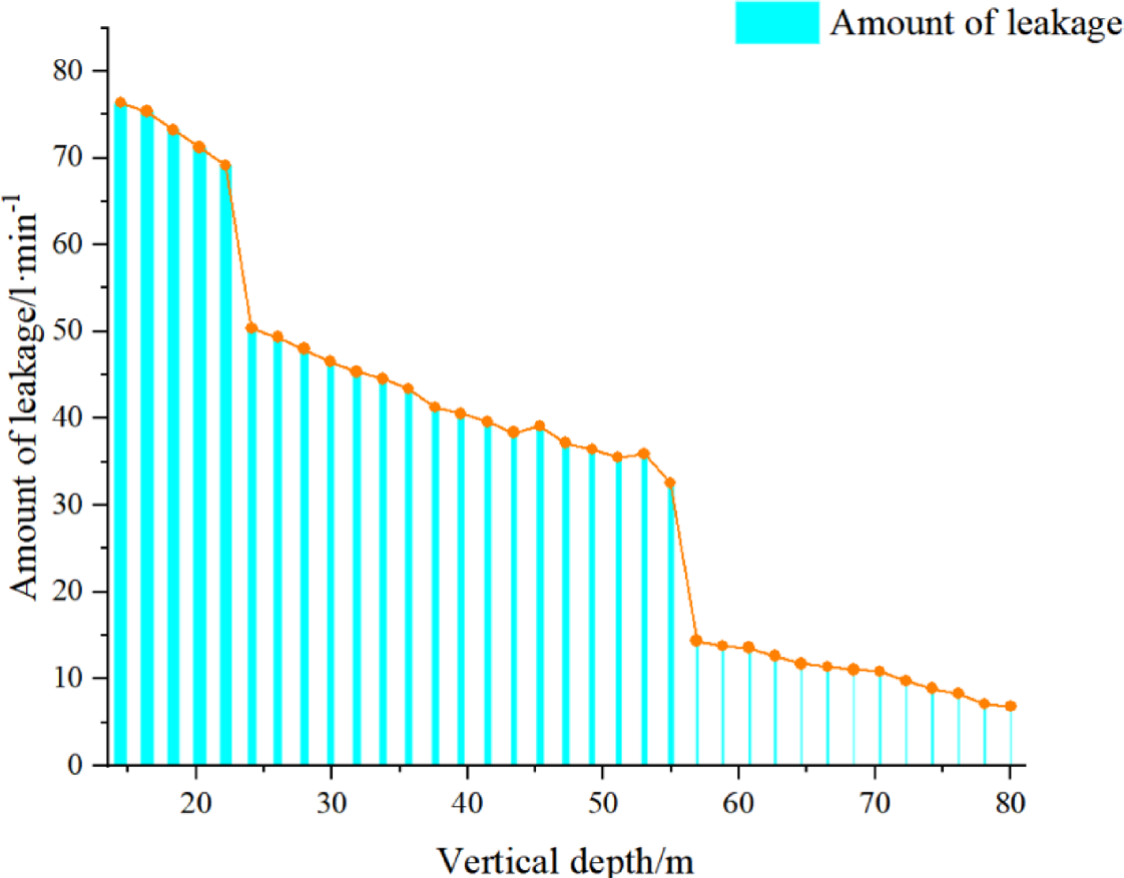
Schematic diagram of leakage and hole depth of No. 3 coal.

According to the mining rules, two boreholes are arranged, and boreholes 1# and 2# are used to measure the 316 working face. The test principle diagram is shown in Fig. 2. The height of the water-conducting fracture zone is determined according to the change in water flow and depth; after sealing the borehole with a sealing capsule, water is injected to observe the leakage change. Judge the height of the fracture zone. The schematic diagram of leakage and hole depth of No. 3 coal is shown in Fig. 3. According to this, it can be concluded that the leakage changes greatly at 52.83 m, so the height of the fracture zone is 52.83 m.

The empirical calculation based on the height of the fracture zone under three is as follows:

$${H_{li}}=\frac{{100\sum M }}{{3.1\sum M +5.0}} \pm 4.0=\frac{{100 \times 7.8}}{{3.1 \times 7.8+5.0}} \pm 4.0=22.7\sim 30.7{\text{m}}$$

At the same time, according to past field experience, when designing and measuring the height of the fracture zone, the empirical calculation value will be expanded by two times, so the empirical calculation result is about 61 m. Compared with the actual value of the height of the water-conducting fracture zone, the prediction results of the empirical formula are seriously distorted. It has been proved that the prediction of fracture zone height when only considering the thickness of the coal seam, has some things that could be improved in the application.

## Analysis of main influencing factors and data sources

### Analysis of main influencing factors

Influencing factors are very important for reasonably predicting fracture zone height. According to scholars’ research, the factors that affect the height development of fracture zones include coal seam dip angle, mining thickness, working face size, mining depth, mining methods and roof management methods, lithology and geological structure, uniaxial compressive strength of roof, time, etc.

When collecting the geological data of some mines and the measured data of the height of the water-conducting fracture zone, by comparing the geological data of the mines, it was found that the geological structure did not affect the height of the water-conducting fracture zone of this working face. The coal seams all belonged to near-horizontal coal seams, and the coal mining methods and roof management methods were the same, both being fully mechanized mining and total collapse method. The geological structure, coal seam dip Angle, coal mining methods and roof management methods of this part of the mines in the collected data do not affect the height of the water-conducting fracture zone. Therefore, the geological structure, coal seam dip Angle, coal mining methods and roof management methods are not analyzed in relation to the height of the water-conducting fracture zone. At this point, considering the quantification of Lithology and formation structure, as well as the uniaxial compressive strength of the roof, the Lithology Proportion Factor (LPF) is taken as one of the input parameters of the intelligent model. This factor is used to quantify the proportional relationship of different lithologies (such as sandstone, mudstone, shale, etc.) in the vertical rock layer profile, in order to characterize the overall failure tendency and structural stability of the rock layer. The essence is the ratio of the height of hard rock to that of the water-conducting fracture zone, with a maximum of 1. The height of the water-conducting fracture zone is mainly affected by the smaller one among the working face sizes. Moreover, in the data collected this time, all the mines are in the direction of long-wall mining, and only the length of the working face is considered.

Therefore, the main factors influencing this analysis are mining thickness, working face length, buried depth, and lithology ratio coefficient.

### Data sources and dataset description

The data used in this article mainly come from two types of channels: one is the data of typical engineering cases publicly published in previous research results, and the other is the measured monitoring and production record data provided by a certain mining area.

Specifically, the collection of some sample data includes the measured and analyzed results of the height of water-conducting fracture zones under different geological conditions in academic literature. These data cover a combination of various mining depths, roof lithology, overlying rock structures and mining methods, and are highly representative. The literature data have undergone screening and standardization processing to ensure the consistency and comparability of variables.

On the other hand, the data provided by the mine mainly comes from the actual mining and excavation working face of a typical coal mining area in North China. It is obtained through geophysical exploration, drilling and hydrogeological testing methods, covering key parameters such as the mining depth, mining height, overlying rock thickness, lithological combination and roof strength of the working face, and also includes the measured height of the corresponding water-conducting fracture zone. This type of data reflects the response characteristics of rock mass under real working conditions, providing a high-quality basis for model training.

The integration of the above two types of data is conducive to constructing a prediction model with good generalization ability, and at the same time enhances the applicability and reference value of the research results in actual engineering scenarios. The specific data table is shown in Table 1.

**Table 1:** Measured value and influencing factor data of water-conducting fracture zone.

ID	Mining height /m	Face length /m	Burial depth /m	Lithology proportion factor	Fracture zone height /m
1	7.5	174	367	0.47	75.5
2	7.5	170	357	0.38	61.9
3	7.5	190	367	0.41	61.8
4	6.1	170	475	0.37	64.6
5	3	186	649	0.23	43
6	5	122	320	0.81	67.7
7	4	175	485	0.36	62.5
8	3.8	168	270	0.65	54.6
9	2.8	156	269	0.68	50.3
10	2.6	147	265	0.6	43.4
11	2.8	148	264	0.26	40.3
12	2.6	168	290	0.37	38.4
13	2.6	168	290	0.18	39.1
14	2.7	192	265	0.56	42.8
15	2.6	185	295	0.64	40.5
16	7	168	433	0.52	73
17	7.4	160	331	0.55	64.2
18	5.3	146	312	0.24	44.2
19	5.7	178	284	0.63	51.4
20	1.9	151	244	0.48	36.8
21	6.6	170	745	0.37	66.6
22	8.1	193	409	0.52	72.9
23	3.9	200	370	0.42	49
24	4.5	135	370	0.45	57.5
25	12.1	220	810	0.43	82.3
26	8.7	198	418	0.45	83
27	7.5	174	367	0.47	75.5
28	8.6	170	357	0.38	66.5
29	8.6	190	367	0.41	61.8
30	8.7	153	434	0.62	71
31	6.5	150	1065	0.63	75.6
32	5.8	154	400	0.81	70.7
33	8	120	272	0.53	62
34	8	170	450	0.55	86.8
35	9.5	123	450	0.65	78
36	4.5	123	450	0.65	49
37	4.7	297	368	0.39	56
38	5.8	178	570	0.34	65.3
39	3.6	146	359	0.21	30
40	3	180	568	0.85	57
41	3	206	516	0.74	54.5
42	2	120	317	0.14	31.61
43	2	120	321	0.16	33
44	2	120	351	0.53	37
45	2	120	358	0.33	34
46	2	120	336	0.12	27.3
47	2	120	363	0.33	31.6
48	3	120	431	0.04	22
49	3.4	120	434	0.46	45
50	2.2	120	413	0.24	35.2
51	2	120	403	0.08	22.6

In order to have a more comprehensive understanding of the distribution characteristics of the input and output data of the model and improve the transparency and repeatability of model construction, this paper conducts statistical description analysis on the datasets used for training and testing.

**Fig. 4:**
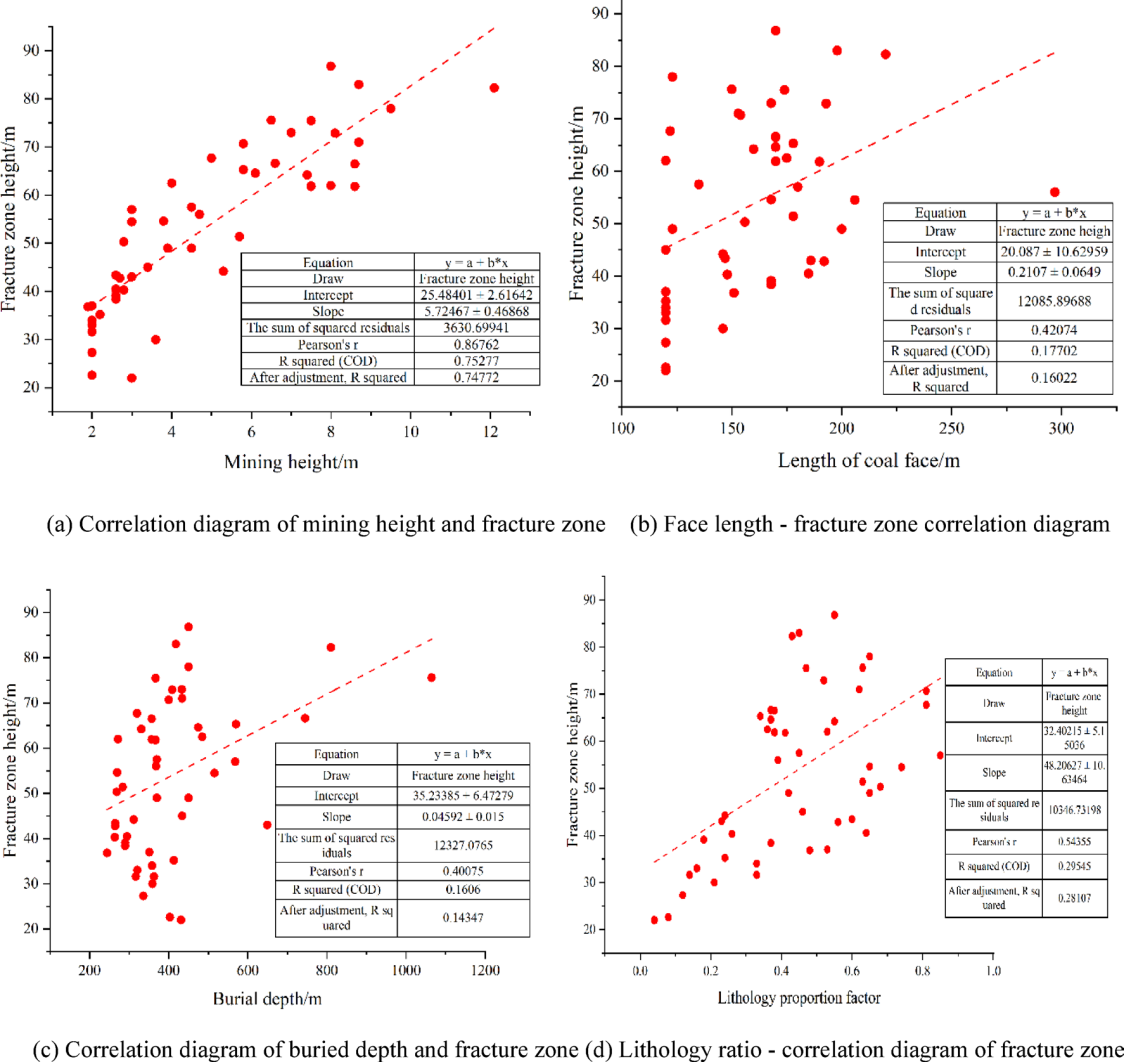
Correlation diagram between influencing factors and water-conducting fracture zone.

Figure 4 lists the basic statistics of each main input feature (such as mining depth, working face length, roof thickness, overburden strength, etc.) and output variable (height of water-conducting fracture zone). It can be significantly seen that the correlation and influence degree of the input features on the output variables.

1. Mining thickness and coal seam mining height are direct factors affecting the development of the water-conducting fracture zone. Within a certain range, the overbearing rock layer gradually breaks as the working face advances, the plastic failure zone of the roof increases, the roof movement value becomes larger and larger, and the height of the water-conducting fracture zone also increases accordingly. It can be known from Fig. 4a that the mining height is significantly positively correlated with the height of the water-conducting fracture zone. Moreover, the mining height ranges from 1.9 to 12.1 m, indicating that the heights of different mining and excavation working faces vary greatly. The influencing factors may be related to the characteristics of the coal seam and the mining process.
2. Working face length: The working face length is an important indicator for measuring the impact of coal seam mining. When the working face is longer, the cantilever support range of the overlying rock in the stope is larger, the self-bearing pressure is greater, and the degree of development of roof rock failure is more complete, which is conducive to the highly developed water-conducting fracture zone. It can be known from Fig. 4b that when the length of the working face increases from 120 to 250 m, the height of the water-conducting fracture zone keeps increasing and shows a positive correlation. Moreover, the variation of the working face length is relatively balanced, with the minimum value being 120 m, the maximum value being 220 m, and the average value being 170.5 m. The standard deviation is 28.3, indicating that the dispersion of the data is moderate.
3. Burial depth: The burial depth of coal seams affects the original stress of the surrounding rock. The variation range of the burial depth is relatively large, ranging from 244 m to 1065 m, and the average value is 406.2 m. The variation of this parameter may be closely related to the geological characteristics and mining conditions of the mining area. It can be known from Fig. 4c that within the range of burial depth less than 600 m, the development height of the water-conducting fracture zone continues to increase. When the burial depth exceeds 600 m, the height of the water-conducting fracture zone will decrease to varying degrees.
4. Lithology proportion coefficient: Under fully mechanized mining conditions, the structural types of the roof rock strata are different, the compressive strength of the overlying rocks is different, and the degree of deformation of the rock strata after mining is different. It can be known from Fig. 4d that the lithology proportional coefficient shows a significant positive correlation with the height of the water-conducting fracture zone. Moreover, the value of the lithology proportional coefficient varies greatly. The minimum value is 0.04, the maximum value is 0.85, the mean value is 0.44, and the standard deviation is 0.22. The fluctuation of this coefficient may reflect the complexity of the physical properties and composition of the rock strata in different working faces.

## Prediction model of fracture zone height

In this study, the prediction models for the water-conducting fracture zone height were developed using Python (version 3.7) in the Jupyter Notebook environment. The following libraries were employed:

Support Vector Regression (SVR): Implemented via the scikit-learn library (sklearn.svm.SVR), with parameters such as kernel type, C, epsilon tuned through grid search combined with 5-fold cross-validation.

Extreme Gradient Boosting (XGBoost): Utilized the xgboost library, with hyperparameters including learning rate, maximum depth, and number of estimators optimized using randomized search and early stopping to prevent overfitting.

Categorical Boosting (CatBoost): Employed the catboost library, suitable for handling categorical features and missing data. Parameters such as depth, learning rate, and iterations were tuned via cross-validation.

The integrated model was constructed by combining the predictions of the above three base models through weighted averaging (or stacking if you used stacking), where weights were determined based on individual model performance on validation data.

For numerical simulation of the fracture zone height, FLAC3D software was used to model the geomechanical behavior of the rock mass under mining conditions. The FLAC3D simulation results served as additional verification and comparison for the machine learning predictions.

All data preprocessing, feature engineering, model training, and evaluation steps were conducted in Python. Performance metrics such as RMSE, R², and MAE were calculated to assess model accuracy.

### Sample data preprocessing

A total of 51 sets of sample data were obtained, and each sample had 5 characteristic values, namely, mining thickness, oblique length, burial depth, lithology ratio coefficient, and fracture zone height. The first four features were input features, and the fifth was the target feature. The ratio of the sample training set to the test set was 8:2.

There are a total of 5 features in the selected dataset. Among them, the first 4 are the input variables of the model, and the fifth one is the prediction target of the model (i.e., the output variable). Due to the different physical meanings of these five features and the significant differences in unit dimensions, directly inputting them into the model can easily lead to some features with a wide range of values dominating the training process, affecting the learning efficiency and prediction accuracy of the model. Therefore, in order to improve the training stability and generalization ability of the model, it is necessary to preprocess the feature data.

In this paper, the Standardization method is adopted to process the first four input features and uniformly convert them into a standard normal distribution with a mean of 0 anda standard deviation of 1. Standardization not only helps alleviate the impact brought by different feature scales, but also better meets the basic assumptions of most machine learning models (such as linear regression, support vector machines, neural networks, etc.) regarding the distribution of input data, such as the assumptions of homoscedasticity and normality. The fifth feature is the target value that the model is to predict. Therefore, it does not participate in standardization processing and keeps its original physical dimension unchanged to ensure the practical significance and interpretability of the prediction results.

Since this study uses a fixed dataset and does not involve new samples in the future, the statistics of the entire training set can be directly utilized during standardization. The standardization method selected is Z-score standardization, and its mathematical expression is:

$${x^ * }=\frac{{x - \mu }}{\sigma }$$

In the formula: ${x^ * }$—Standardized eigenvalues, *x*—Original eigenvalue, $\mu$—The mean value of this feature, $\sigma$—The standard deviation of this feature

**Fig. 5:**
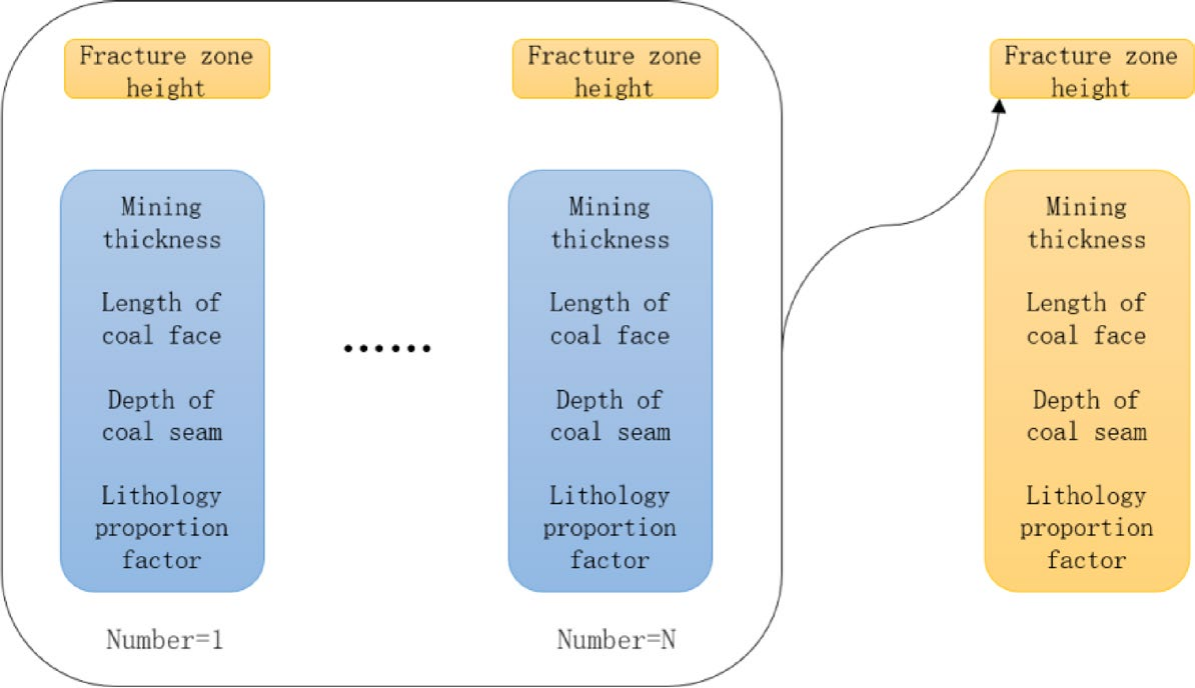
Schematic diagram of “feature parameter - label”.

The models trained in this paper are all supervised learning models, so the data are trained according to the corresponding form of “feature parameter - label”, and the training samples are generated, as shown in Fig. 5. Data with similar conditions are collected by referring to the research working face information to predict the height of the fracture zone.

### Hyperparameter selection and optimization

Before training the model, it is necessary to confirm the parameters of the model first. Model parameters come in two forms: internal parameters and hyperparameters. Internal parameters are unalterable modular independent variables and are part of machine learning itself. Hyperparameter optimization is an external configuration variable of the model, usually set artificially, and the setting of hyperparameters has a significant relationship with the accuracy rate of the model. Meanwhile, when the model is overfitted or underfitted, it is also necessary to adjust the hyperparameters to make the model reach a reasonable level. As shown in Table 2, these are some hyperparameters set for the selected model in this paper.

**Table 2:** Model hyperparameters.

Parameters	Meaning
random_state	Set the seed of the random number generator to control randomness
depth	The maximum depth of the decision tree limits the number of splits of the tree
learning_rate	Learning rate, controlling the contribution weight of each tree to the final prediction result
iterations	The number of iterations of model training
l2_leaf_reg	The coefficients of the L2 regularization term act on the weights of the leaf nodes

In response to the problem that some hyperparameter selections are not reasonable enough, resulting in unsatisfactory model effects, some scholars have proposed the method of hyperparameter optimization, adjusting the hyperparameters to achieve the optimal effect of the model. Common parameter optimization methods include grid search and Bayesian optimization algorithms. Grid search is essentially an enumeration method. This method is inefficient: it needs to traverse all parameter combinations, and the computational cost increases exponentially with the number of parameters. However, Bayesian optimization algorithms reduce the number of evaluations through intelligent sampling and usually only require 10–20% of the number of iterations of grid search to converge. Focus on high-potential areas, avoid ineffective experiments, significantly save computing resources, dynamically adjust the search direction, and adapt to the shape of the objective function. Therefore, this paper selects the Bayesian optimization method to optimize the hyperparameters.

### K fold cross-validation

**Fig. 6:**
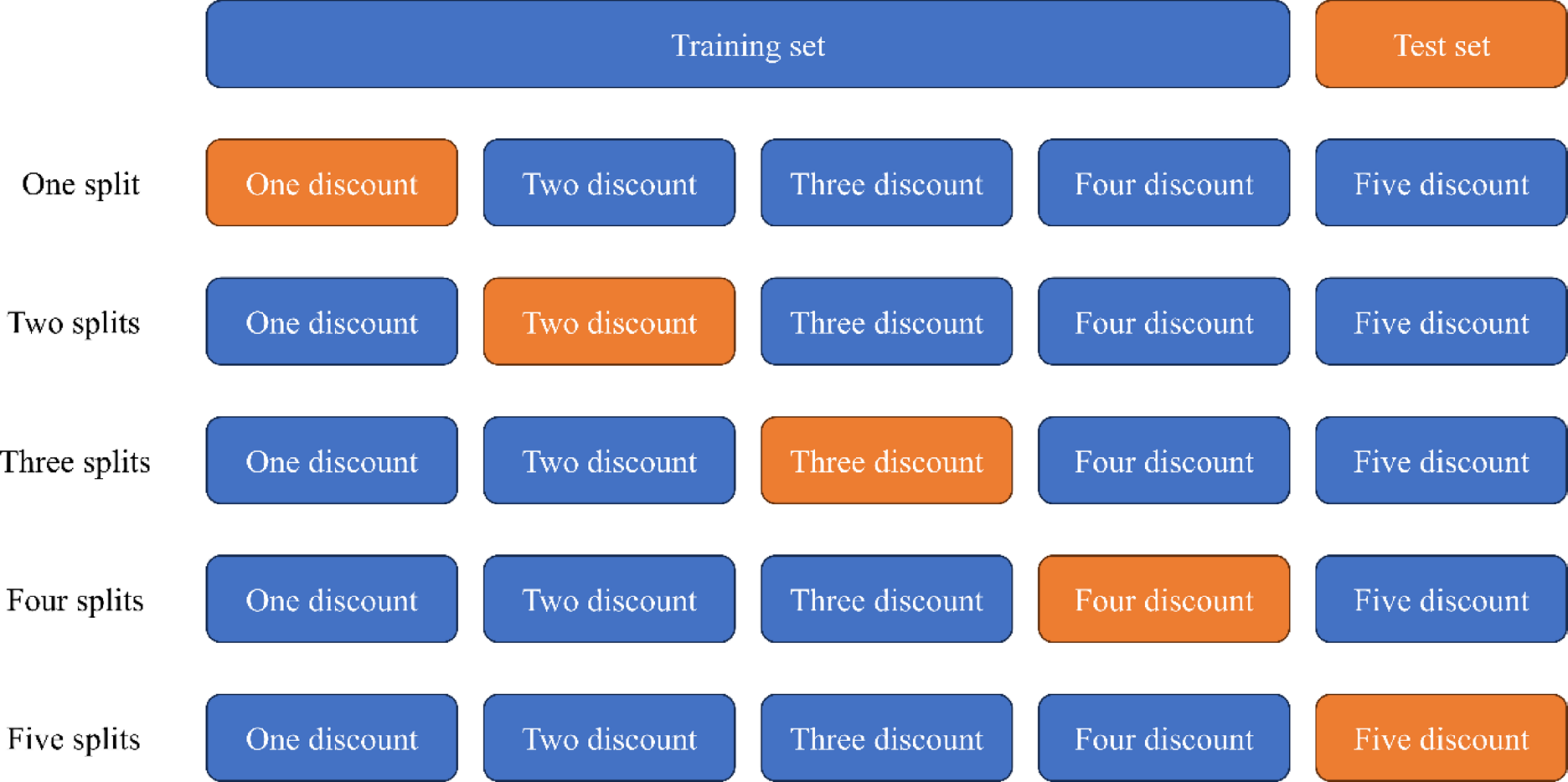
Cross-validation.

k-Fold Cross-Validation is a statistical method used to evaluate the generalization ability of machine learning models. As shown in Fig. 6. The core idea is to divide the original data set into K mutually exclusive subsets of similar size (referred to as “folding”). Each time, one of the subsets is used as the test sample data, and the remaining K-1 subsets are combined as the training sample data. This process is repeated K times for training and validation. This process is a test. After testing the model once, the selected sample data is replaced. Select the new sample data as the new test sample data and repeat K times. Finally, the average value of K evaluation results is taken as the estimation of the model performance. This method makes full use of the data and employs non-repetitive sampling of the original data set. During each iteration, each sample has only one chance to be classified as either the learning set or the test set, reducing the random influence of a single data partition and enhancing the stability and reliability of the model evaluation.

### Model evaluation indicators

The performance evaluation indicators of the model can directly reflect the superiority or inferiority of the model. MSE is the average of the squares of the differences between the predicted values and the true values, reflecting the overall error size of the model’s prediction. Essentially, it is the sum of the variance of the prediction error and the square of the deviation. Its minimization process is equivalent to finding the optimal unbiased estimation. RMSE is the square root of MSE, which restores the error to the original data dimension and more intuitively reflects the average deviation degree between the predicted value and the true value. It is the standardized version of MSE. R² measures the model’s ability to interpret the variance of the data, that is, the degree of improvement in the model’s prediction compared to simply using the mean prediction. The essence is the square of the correlation between the predicted value and the true value. In linear regression, R^2^ = r^2^, where r is the Pearson correlation coefficient. MARE is the average of the absolute percentage of the prediction error relative to the true value, eliminating the influence of dimensions, reflecting the relative size of the prediction error, and treating the errors of high-value samples and low-value samples equally. SI is the ratio of RMSE to the mean of true values, expressed as a percentage of the dispersion of prediction errors, and is suitable for datasets with high volatility. NSE is a commonly used indicator in hydrological models, reflecting the degree of improvement in model prediction compared to directly using the mean. However, its formula is the same as R2, emphasizing the model’s ability to capture the dynamics of hydrological processes. PBIAS represents the systematic deviation direction of the predicted value relative to the true value, and the positive or negative sign of PBIAS indicates the deviation direction. PBIAS > 0 indicates that the model underestimates the true value, and PBIAS < 0 indicates that the model overestimates the true value, clearly indicating the overall bias trend of the model. The core logic of using the above evaluation metrics is that MSE/RMSE provides absolute error, R²/NSE assesses the optimization potential of the model, MARE/SI eliminates dimensional influence, and PBIAS captures systematic bias.

1 $${\text{MSE}} = \frac{1}{n}\mathop \sum \limits_{{i = 1}}^{n} (y_{i} - \hat{y}_{i} )^{2}$$

2 $${\text{RMSE}} = \sqrt {{\text{MSE}}} = \sqrt {\frac{1}{n}\mathop \sum \limits_{{i = 1}}^{n} (y_{i} - \hat{y}_{i} )^{2} }$$

3 $$R^{2} = 1 - \frac{{\sum\nolimits_{{i = 1}}^{n} {(y_{i} - \hat{y}_{i} )^{2} } }}{{\sum\nolimits_{{i = 1}}^{n} {(y_{i} - \bar{y})^{2} } }}$$

4 $${\text{MARE}} = \frac{1}{n}\mathop \sum \limits_{{i = 1}}^{n} \left| {\frac{{y_{i} - \hat{y}_{i} }}{{y_{i} }}} \right|$$

5 $${\text{SI}}=\frac{{{\text{RMSE}}}}{{\bar {y}}} \times 100{{\% }}$$

6 $${\text{NSE}} = 1 - \frac{{\sum\nolimits_{{i = 1}}^{n} {(y_{i} - \hat{y}_{i} )^{2} } }}{{\sum\nolimits_{{i = 1}}^{n} {(y_{i} - \bar{y})^{2} } }}$$

7 $${\text{PBIAS}}=\frac{{\sum\nolimits_{{i=1}}^{n} {({y_i} - {{\hat {y}}_i})} }}{{\sum\nolimits_{{i=1}}^{n} {{y_i}} }} \times 100{{\% }}$$

In the formula: ${y_i}$—The true value of the *i*th sample; ${\hat {y}_i}$—The predicted value of the *i*th sample; *n*—Total number of samples; $\bar {y}$—The mean value of the true value

### Model selection and integration strategy

To achieve high-precision prediction of the height of the water-conducting fracture zone in coal mines, the selection of the model is of crucial importance. Due to the limited sample size of the dataset adopted in this study and the nonlinear coupling relationship among each input variable, the model not only needs to have good nonlinear modeling ability, but also should have strong adaptability, robustness and high prediction accuracy for small sample data.

#### Model selection principles

Comprehensively considering the following several factors, determine the base learner and meta-learner used to construct the integrated model in this study:

1. High prediction accuracy: It can accurately capture the complex nonlinear relationship between the height of the water-conducting fracture zone and the influencing factors;
2. Friendly to small sample data: It can achieve relatively stable training and generalization under the condition of limited sample size;
3. Moderate computational efficiency and parameter tuning difficulty: The model training speed is fast, and the difficulty of parameter optimization is relatively low;
4. Possess interpretability or feature weight analysis capabilities: It helps to understand the influence of each input factor on the prediction results;
5. Compatible with integrated frameworks and supporting stacked structures: facilitating the fusion of multiple models, enhancing overall robustness and generalization capabilities.

#### Model composition and integration structure

Based on the above principles, this paper finally selects Support Vector Regression (SVR) and Extreme Gradient Boosting (XGBoost) as the basic learners, and CatBoost as the meta-learner. Construct a Stacking ensemble model for predicting the height of water-conducting fracture zones. The characteristics of each model are as follows:

1. SVR: Simple structure and strong nonlinear fitting ability.
  SVR is an extension of Support Vector Machine in regression problems and can effectively handle problems with strong nonlinear relationships and small sample sizes. Compared with artificial neural networks (ANN), SVR is insensitive to the distribution of input data and the setting of hyperparameters, and the training process is more stable. It is especially suitable for small sample prediction problems with non-stationing.
2. XGBoost: An efficient and robust integrated tree model.
  XGBoost is currently a superior gradient boosting decision tree model with excellent modeling capabilities and automatic feature selection capabilities. Compared with methods such as GEP (Gene Expression Programming) and GPR (Gaussian Process Regression), XGBoost has higher training efficiency, stronger robustness, and can effectively alleviate the problem of overfitting. Its built-in feature importance assessment function also helps to improve the interpretability of the model.
3. CatBoost: As a meta-model, it further optimizes the prediction performance.
  CatBoost is a boosting algorithm based on a symmetric tree structure, featuring strong generalization ability and excellent convergence performance. As a meta-learner, it can relearn and integrate the prediction results of multiple basic models for optimization. It has relatively low requirements for data preprocessing and can enhance the stability and overall performance of the integrated structure.

To verify the rationality of the model selection, this paper also comprehensively analyzes other advanced methods commonly used in geological modeling and prediction at present. Such as ANN, GEP, GPR, Stochastic Gradient Boosting (SGB), Natural Gradient Boosting (NGB), and various evolutionary algorithms (such as genetic algorithm, particle swarm optimization, etc.). The results show that although these methods have certain advantages, there are specific limitations in practical applications: ANN: Sensitive to sample size and parameters, prone to fall into local optimum, unstable training process, and poor interpretability. GEP: It has complex expression, poor result stability, high computing resource requirements, and is not easy to integrate. GPR: It has high computational complexity and limited modeling capabilities for large-scale or high-dimensional data. NGB: The model improvement effect is limited, the training time is long, and it is not easy to integrate into the stacked structure.

Comprehensively considered, by fusing the prediction results of SVR and XGBoost through the Stacking Ensemble method and constructing the meta-learner with CatBoost, the modeling differences of different models on the data can be fully utilized to achieve performance complementarity. This integrated structure can not only improve the overall prediction accuracy of the model, but also enhance its stability and generalability in practical engineering applications.

### SVR model prediction

#### SVR regression prediction principle

Support vector machine regression (SVR) is a method to deal with regression prediction problems. Its principle is to use the kernel function to map nonlinear problems in low-dimensional space to linear problems in high-dimensional space for regression solutions. That is, using the known training sample set (x_i_, y_i_), i = 1,2,…, n, x_i_∈R_n_, y∈R, find the mapping relationship between y and x:

8 $$y = \omega \varphi (x)+b$$

Where: $\omega$ is the weight vector; $\varphi (x)$ is a function that maps the input space to the high-dimensional feature space; b- Indicates the threshold.

The solution of formula (8) can be estimated using the following formula:

9 $$R(\omega )=\hbox{min} \left[ {\frac{1}{2}||\omega |{|^2}+C\sum\limits_{{i=1}}^{n} {({\xi _i}+{\xi _i}^{*})} } \right]$$

The constraint conditions are:

10 $$\left\{ {\begin{array}{*{20}c} {y_{i} - [\omega \varphi (x_{i} ) + b]\leq \varepsilon + \xi _{i} } \\ {\omega \varphi (x_{i} ) + b - y_{i}\leq \varepsilon + \xi _{i}^{ * } } \\ {\xi _{i} ,\xi _{i}^{ * } \geq 0} \\ \end{array} } \right.$$

Where: $\varepsilon$ is the insensitive loss function parameter; $\xi$ and $\xi^{ * }$is the relaxation variable; C is a penalty parameter whose function is to regulate empirical risk and model complexity.

By introducing Lagrange function, the dual problem of formula (10) is obtained:

11 $$\begin{array}{*{20}c} {J\left( {\alpha _{i} ,\alpha _{i}^{ * } } \right) = \max \left[ {\frac{1}{2}\mathop \sum \limits_{{i = 1}}^{n} \mathop \sum \limits_{{j = 1}}^{n} \left( {\alpha _{i} - \alpha _{i}^{ * } } \right)\left( {\alpha _{j} - \alpha _{j}^{ * } } \right)} \right]} \\ { - K\left( {x_{i} ,x_{j} } \right) + \mathop \sum \limits_{{i = 1}}^{n} \alpha _{i}^{ * } \left( {y_{i} - \varepsilon } \right) - \mathop \sum \limits_{{i = 1}}^{n} \alpha _{i} \left( {y_{i} - \varepsilon } \right)} \\ \end{array}$$

The constraint conditions are:

12 $$\mathop \sum \limits_{{i = 1}}^{n} \left( {\alpha _{i} - \alpha _{i}^{ * } } \right) = 0\quad 0 \le \alpha _{i} ,\alpha _{i}^{ * } \le C$$

Where:,${\alpha _i}$,$\alpha _{i}^{ * }$ is the Lagrange multiplier; $K({x_i},{x_j})$ is the kernel function. Radial basis kernel function is selected:

13 $$K({x_i},{x_j})=\exp \left[ { - \frac{{{x_i} - {x_j}^{2}}}{{2{\sigma ^2}}}} \right]$$

Where: $\sigma$is the kernel function parameter.

Finally, the regression function of support vector machine is obtained:

14 $$y = \mathop \sum \limits_{{i = 1}}^{n} (\alpha _{i} - \alpha _{i}^{ * } )K(x_{i} ,x_{j} ) + b$$

#### SVR model construction

**Fig. 7:**
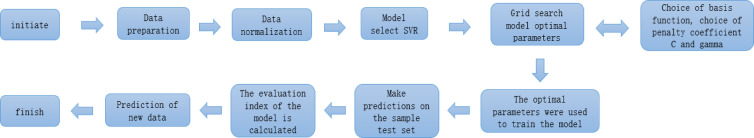
SVR model construction flow chart.

The pre-processed feature data were distributed between [0,1], and the support vector regression (SVR) model was selected as the prediction model. To choose the optimal combination of model parameters, a grid search is used to optimize SVR model parameters. Different kernel functions (such as radial basis kernel function), different penalty coefficients C and different kernel parameters γ are tried to improve the performance and prediction accuracy of the model. Then, the model is trained using the training data, and the test set is used for prediction. The root mean square error (RMSE), R-squared coefficient and other evaluation indexes of the model are calculated. Finally, the trained SVR model is used to predict the new data, and the expected value of the height of the coal mine water conduction fracture zone is obtained. The model construction process is shown in Fig. 7.

#### Model detection

**Fig. 8:**
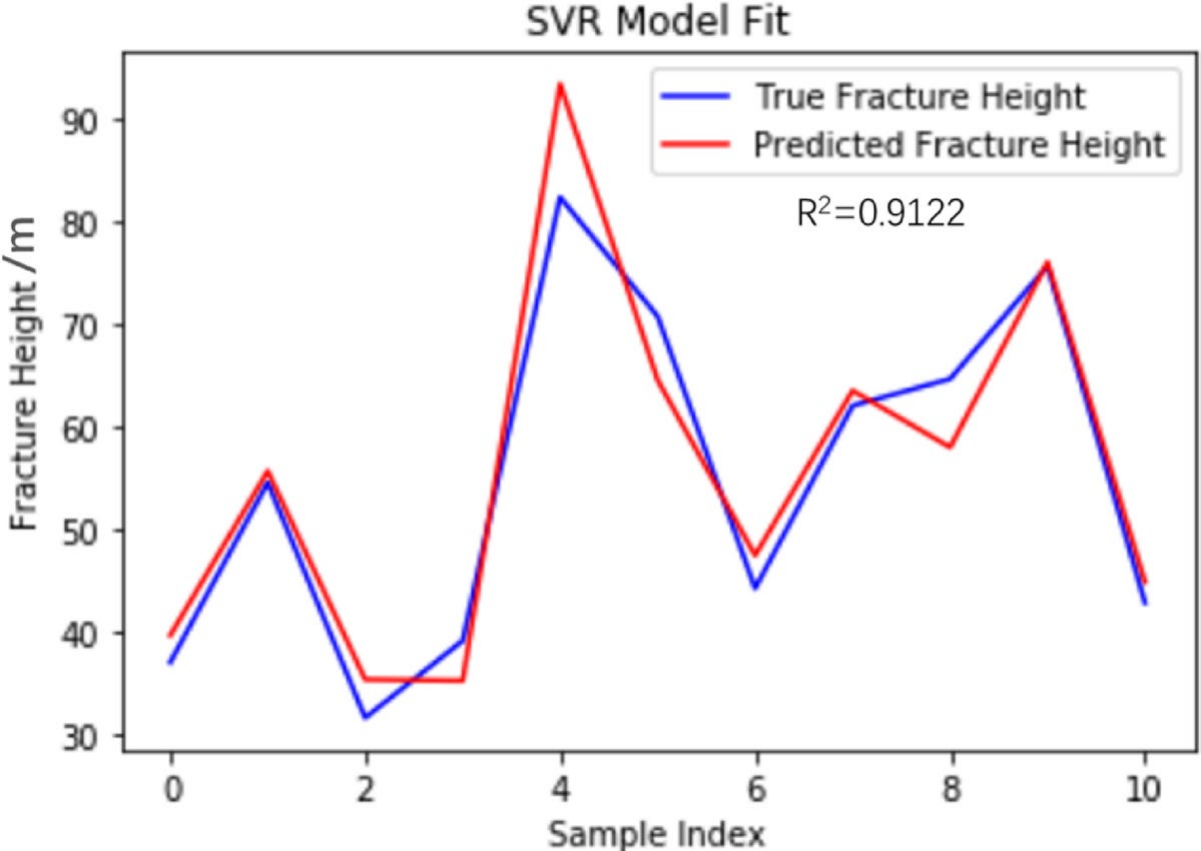
SVR model fitting diagram.

The hyperparameters are optimized through the Bayesian optimization algorithm. When the SVR model uses the Radial Basis function (RBF) kernel, the penalty coefficient C = 500 and gamma = 0.01, the model performance reaches the optimal state. As the basic model, the SVR model also only calculates two evaluation indicators, namely the root mean square error (RMSE) and the goodness of fit (R^2^), for the comparison of the prediction effects among multiple subsequent models. Substituting into the SVR regression model, training and testing were conducted using the data in Table 1. Based on the SVR model to predict the height value of the fracture zone, R^2^ and RMSE were 0.9122 and 4.84 respectively. The fitting graph of the prediction results of the SVR model and the true values is shown in Fig. 8. The predicted height of the water-conducting fracture zone in the No. 3 coal seam of Husheng Coal Mine is 58.14 m. The relative error rate compared with the actual fracture zone height is 10.05%.

To ensure the validity of the test results, cross-validation was conducted using the same model parameters. 20% of the data was used as the test set, and the remaining 80% of the data was used as the training set. Table 3 shows the accuracy of each prediction model for cross-validation training. Finally, the average prediction accuracy of the test set was 96.6%, which proved the prediction ability of the SVR model.

**Table 3:** Cross-validation results of the SVR model.

Fold	Accuracy
1	95%
2	100%
3	100%
4	95%
5	95%
Average	96.6%

### XGBoost model prediction

#### XGBoost prediction principle

**Fig. 9:**
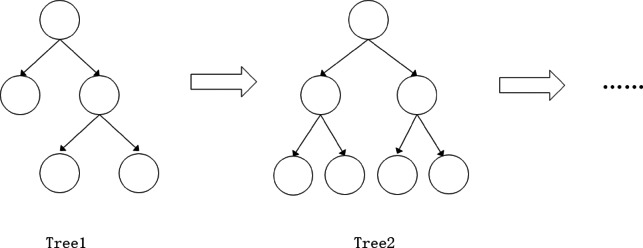
XGBoost diagram.

The full name of XGBoost is eXtreme Gradient Boosting, and its Chinese name is Extreme Gradient Boosting Tree. Xgboost is an extension of the Gradient Boosting Machine algorithm and the realization of a gradient boosting machine, which can fully use the computer’s central processor to compute massive data in parallel. The prediction accuracy of the model is higher. Through the comprehensive prediction of multiple weak learners, the model cannot easily overfit and can handle high-dimensional sparse features. Therefore, the XGBoost model has higher operational efficiency compared with similar algorithms. The structure of XGBoost is shown in Fig. 9.

The XGBoost algorithm works as follows:

The objective function is formula (8):

15 $$\left\{ {\begin{array}{*{20}c}{L = \mathop \sum \limits_{i} l(\hat{y}_{i} ,Y_{i} ) + \sum {{\Omega }}(f_{k} )} \\ {{{\Omega }}(f_{k} ) = \gamma T + \frac{1}{2}\lambda \parallel {\mathbf{w}}\parallel ^{2} }\end{array} }\right.$$

In Eq. (15), l is a loss function. For classification or regression tasks, different loss functions are selected to measure the difference between the predicted value $\hat {y}_{i}^{{}}$ and the true value ${Y_i}$.${\text{\varvec{\Omega}}}$ is the regular term of the model, used to inhibit overfitting of the model. $\gamma$ is the complexity parameter, T is the number of nodes in the middle of the tree, and $\lambda$ is the penalty coefficient of leaf weight ${\mathbf{w}}$. The complete iteration formula for XGBoost is formula (9).

16 $$\hat {y}_{i}^{{k+1}}=\hat {y}_{i}^{k}+\eta {f_{k+1}}({X_i})$$

In Eq. (16), ${f_{k+1}}$ is the model of the k + 1 tree and $\eta$ is the learning rate, which belongs to one of the many parameters of XGBoost. Since the model contains many parameters, manual adjustment is time-consuming and laborious, this paper uses grid search to determine the optimal parameter combination of the model. The instantiation in Eq. (16) ${\hat {y}_i}$ is carried out to represent the predicted value of the i th instance in the t iteration and the t th tree established, ${f_t}$ represents the t th tree built, so Eq. (10) is obtained.

17 $$\begin{array}{*{20}c} {L^{t} = \mathop \sum \limits_{{i = 1}}^{n} l(y_{i} ,\hat{y}_{i}^{{t - 1}} + f_{t} (X_{i} )) + {{\Omega }}(f_{t} )} \\\quad { \approx \mathop \sum \limits_{{i = 1}}^{{\text{n}}} [g_{i} f_{t} (X_{i} ) + \frac{1}{2}h_{i} f_{t}^{2} (X_{i} )] + {{\Omega }}(f_{t} )} \\ \end{array}$$

In Eq. (17), ${g_i}$ and ${h_i}$ is the first and second order Taylor expansion of the loss function, respectively. Defined ${I_j}=\{ i|q({X_i})=j\}$as a set of instances of leaves j, Eq. (17) can be written as Eq. (19).

18 $$L^{t} = \mathop \sum \limits_{{i = 1}}^{{\text{n}}} \left[g_{i} f_{t} (X_{i} ) + \frac{1}{2}h_{i} f_{t}^{2} (X_{i} )\right] + \gamma T + \frac{1}{2}\lambda \mathop \sum \limits_{{j = 1}}^{T} w_{j}^{2}$$

19 $$L^{t} = \mathop \sum \limits_{{j = 1}}^{T} \left[\left(\mathop \sum \limits_{{i \in I_{j} }} g_{i} \right)w_{j} + \frac{1}{2}\left(\mathop \sum \limits_{{i \in I_{j} }} h_{i} + \lambda \right)w_{j}^{2} \right] + \gamma T$$

When q(X) is determined, based on the function extremum problem, the optimal weight $w_{j}^{ * }$ of the middle node j of the tree model and the optimal value of the objective function can be obtained as Eq. (21).

20 $$w_{j}^{ * } = \frac{{\mathop \sum \nolimits_{{i \in I_{j} }} g_{i} }}{{\mathop \sum \nolimits_{{i \in I_{j} }} h_{i} + \lambda }}$$

21 $$L^{t} (q) = - \frac{1}{2}\mathop \sum \limits_{{j = 1}}^{T} \frac{{(\mathop \sum \nolimits_{{i \in I_{j} }} g_{i} )^{2} }}{{\mathop \sum \nolimits_{{i \in I_{j} }} h_{i} + \lambda }} + \gamma T$$

#### XGBoost model construction

**Fig. 10:**
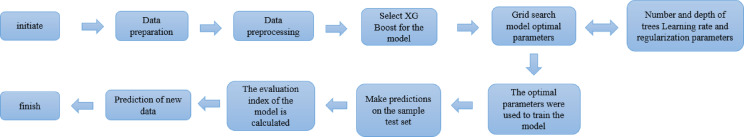
Flowchart of XG Boost model construction.

After the feature data is preprocessed, it is distributed between [0,1], and the extreme gradient Lift tree (XGboost) model is selected as the prediction model. A grid search approach can be used to optimize the XGBoost model and find the best combination of hyperparameters. By trying different combinations of hyperparameters, including the number and depth of the tree, the learning rate, and the regularization parameters, the performance and prediction accuracy of the model can be improved through this systematic approach. After completing the grid search and selecting the best combination of hyperparameters, the XGBoost model can be trained using the training data. Then, the test set can be used to calculate the root mean square error (RMSE), R-squared coefficient, and other model evaluation indicators and quantify the model’s prediction ability. Finally, the trained XGBoost model is used to predict the new data, and the expected height of the coal mine water conduction fracture zone is obtained. The model construction process is shown in Fig. 10.

#### Model detection

**Fig. 11:**
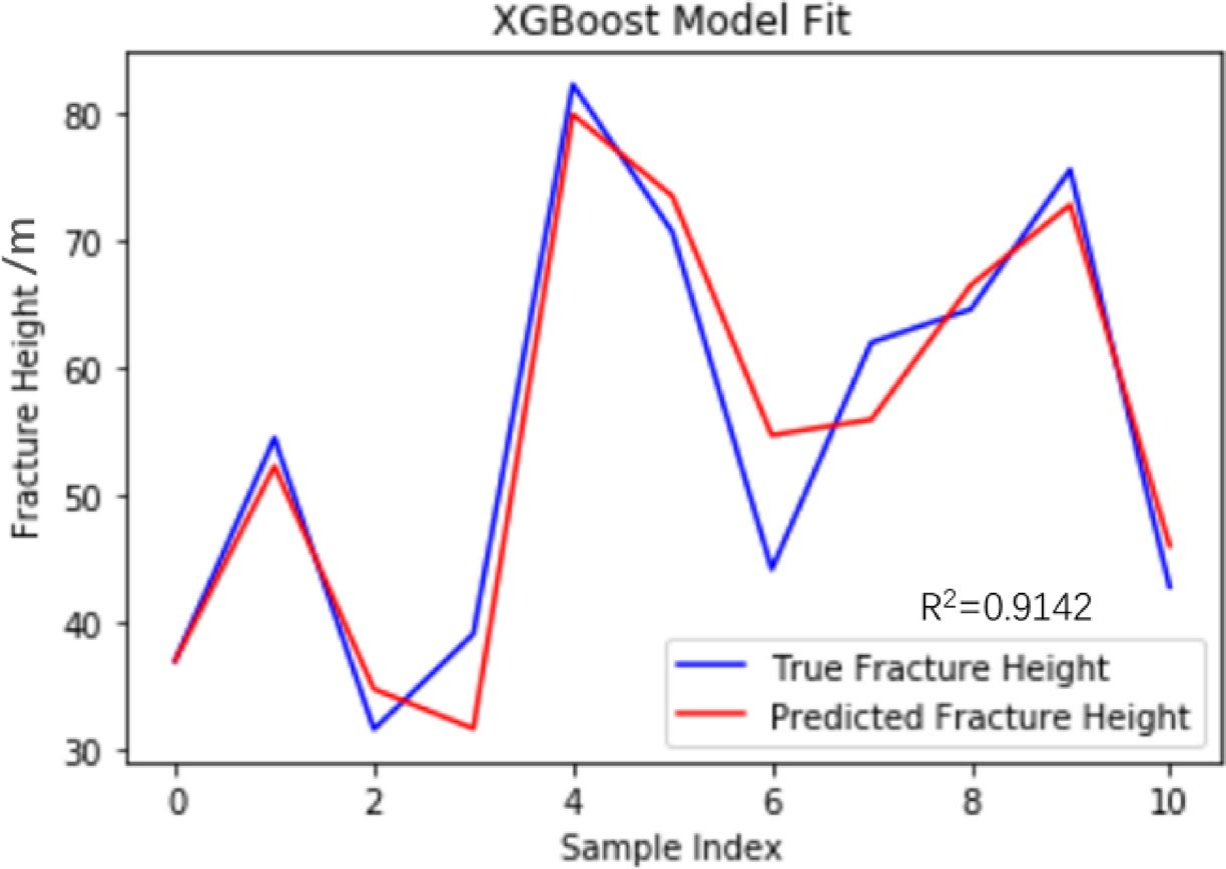
XGBoost model fitting diagram.

The hyperparameters are optimized through the Bayesian optimization algorithm. When the XGBoost model uses 500 trees, has a learning rate of 0.01 and a maximum depth of 3, the model performance reaches the optimal state. As a basic model, the XGBoost model also only calculates two evaluation metrics, RMSE and R^2^, to compare the prediction effects among multiple subsequent models. Substituting into the XGBoost model, training and testing were conducted using the data in Table 1. Based on the XGBoost model to predict the height value of the fracture zone, the goodness of fit R^2^, mean square error (MSE), and root mean square error (RMSE) were 0.9142 and 4.79 respectively. The fitting graph of the prediction results of the XGBoost model and the true values is shown in Fig. 11. The predicted height of the water-conducting fracture zone in the No. 3 coal seam of Husheng Coal Mine is 56.615 m. The relative error rate compared with the actual fracture zone height is only 6.28%.

To ensure the validity of the test results, cross-validation was conducted using the same model parameters. 20% of the data was used as the test set, and the remaining 80% of the data was used as the training set. Table 4 shows the accuracy of the model trained by cross-validation. Finally, the average prediction accuracy of the test set is 97%, which indicates that the XGBoost model has a good prediction ability.

**Table 4:** Cross-validation results of the XGboost model.

Fold	Accuracy
1	99%
2	96%
3	97%
4	98%
5	95%
Average	97%

### Ensemble learning model prediction

Based on the previous SVR and XGBoost model analysis results, stack integration technology is adopted to improve prediction accuracy further, and CatBoost is introduced as a metamodel. Stack integration is a powerful machine learning technique that combines the predictions of multiple base models to create a more powerful prediction model.

In the previous analysis, support vector back (SVR) and XGBoost models were used to predict the height of the coal mine water conduction fracture zone. Now, the predictions from these models are fed into the CatBoost metamodel as new features. CatBoost is an advanced gradient-enhanced decision tree algorithm that handles categorical variables and captures nonlinear patterns.

In a stacked integration framework, the CatBoost metamodel learns predictions for the best combination of SVR and XGBoost. It can capture patterns the underlying model might miss and exploit them. CatBoost can identify and leverage the complementarity between SVR and XGBoost to provide more accurate final predictions.

#### Prediction principle of ensemble learning model

The principle behind ensemble learning is that combining multiple models is often more powerful than a single model. Each base model can capture different aspects and patterns of the data. By stacking their predictions, the CatBoost metamodel can learn how to weigh and combine them to produce more accurate results.

CatBoost is particularly well suited for dealing with categorical variables, which are important for coal mine forecasting scenarios. Categorical variables represent categories or groups. CatBoost has built-in functionality to handle such variables and extract patterns from them.

Multiple powerful and complementary algorithms can be leveraged by taking a stackable integration approach and introducing CatBoost. SVR can capture nonlinear patterns in the data, XGBoost can handle complex feature interactions, and CatBoost can integrate their predictions and learn how to combine them best.

Using ensemble learning models, the advantages of stacked integration frameworks are their robustness and generalization. By leveraging multiple models, the risk of overfitting can be reduced, and the model’s performance on unknown data can be improved. The CatBoost metamodel is the final decision maker, leveraging knowledge of the underlying model to make more accurate predictions.

The main considerations for introducing CatBoost as a metamodel are as follows:

(1) Ordered target coding.

CatBoost uses an ordered target encoding feature to process category features efficiently without data leakage. This makes CatBoost perform well when dealing with high-dimensional sparse features, especially for complex regression problems such as fracture zone prediction. The formula for ordered object coding is as follows:

22 $$\mathop {Encoding(x_{i} )} = \frac{{\mathop \sum \nolimits_{{j|x_{j} - x_{i} }} y_{j} }}{{\mathop \sum \nolimits_{{j|x_{j} - x_{i} }} w_{j} }} - \frac{{\mathop \sum \nolimits_{j} y_{j} }}{{\mathop \sum \nolimits_{j} w_{j} }}$$

Where ${x_i}$ is the value of the class feature, ${y_j}$ is the target value, and ${w_j}$ is the sample weight. This encoding method can effectively capture the relationship between category features and target values without data leakage.

(2) Gradient Lifting Decision Tree (GBDT).

Similar to XGBoost, CatBoost is also based on the GBDT algorithm. Still, it has made several improvements to the original algorithm, such as ordered gradient lifting, leaf growth strategy, etc., to improve the accuracy and training speed of the model. As a metamodel, CatBoost does a good job of capturing nonlinear relationships between the underlying models.

CatBoost’s GBDT algorithm can be expressed as follows:

23 $$F(x) = \mathop \sum \limits_{{k = 1}}^{K} f_{k} (x)$$

Where $F(x)$ is the final prediction model, which ${f_k}(x)$ is the K_th_ decision tree. GBDT is constantly adding new decision trees so that the prediction results are moving in the right direction at each step.

(3) Overfitting prevention and control.

CatBoost has built a variety of overfitting prevention and control mechanisms, such as stochastic gradient weighting, leaf growth control, etc., to make the model more robust when fitting complex data and avoid overfitting. This is especially important for metamodels that integrate multiple base models to prevent the integrated model from focusing too much on certain features at the expense of other important features.

The leaf growth strategy adopted by CatBoost is called “Ordered Uplifting,” and the formula is as follows:

24 $$Score(a,b)=\frac{1}{{{W_a}+{W_b}}}\left( {\frac{{{W_a}{W_b}}}{{{W_a}+{W_b}}}{{\left( {\frac{{{S_a}}}{{{W_a}}} - \frac{{{S_b}}}{{{W_b}}}} \right)}^2}+\alpha \cdot (K - 1)} \right)$$

Where a and b represent two leaf nodes respectively, ${W_a}$ and ${W_b}$ are the sum of their weights, ${S_a}$ and ${S_b}$ are the statistics of the target values, and K is the number of leaf nodes, and $\alpha$ is a regularization parameter. This strategy can better control the complexity of the model and prevent overfitting.

The random gradient weighting introduced by CatBoost is as follows:

25 $${w_i}=\frac{1}{{1+exp( - {z_i})}}$$

Where ${z_i}$ is a random number sampled from a standard Gaussian distribution and ${w_i}$ is the weight of the i th sample. This approach can reduce the impact of some gradients, thereby reducing the risk of overfitting.

(4) Parallel computing.

CatBoost supports multi-thread parallel processing, which can fully utilize the multi-core advantage of modern CPUs and speed up the model’s training speed. This is especially critical for training complex integrated models and can significantly reduce training time.

(5) Automatic feature combination.

CatBoost has automatic feature combination capabilities that automatically generate new combined features based on training data and capture higher-order feature interactions. This is very helpful for integrating the predictions of multiple base models into the metamodel as a new feature input, which can improve the metamodel’s expression ability.

#### Ensemble learning model construction

After the SVR and XGBoost models are trained, they are used to predict the test set’s results, and the SVR and XGBoost predictions are stacked to form a new feature matrix, svm_xgb_pred.

**Fig. 12:**
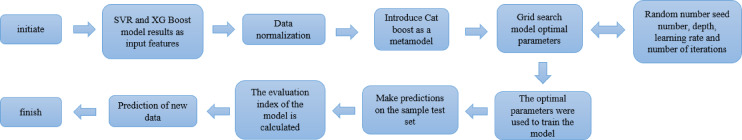
Flowchart of integrated learning model construction.

The data is preprocessed to ensure that all features have been normalized to the interval of [0,1] to guarantee the effectiveness of model training. Support Vector regression (SVR) and XGBoost are selected as the base models in the ensemble learning model, and CatBoost is selected as the meta-model. The hyperparameters are optimized through the Bayesian optimization algorithm, including the number of random number seeds, depth, learning rate and the number of iterations. After obtaining the optimal parameters, the integrated model is trained, and then the test set is used for prediction to calculate model evaluation indicators such as mean square error (MSE), root mean square error (RMSE), and R-squared coefficients. Finally, the trained CatBoost model is used to predict the new data, and the predicted value of the integrated model for the height of the water-conducting fracture zone is obtained. The flowchart of model construction is shown in Fig. 12.

#### Model detection

**Fig. 13:**
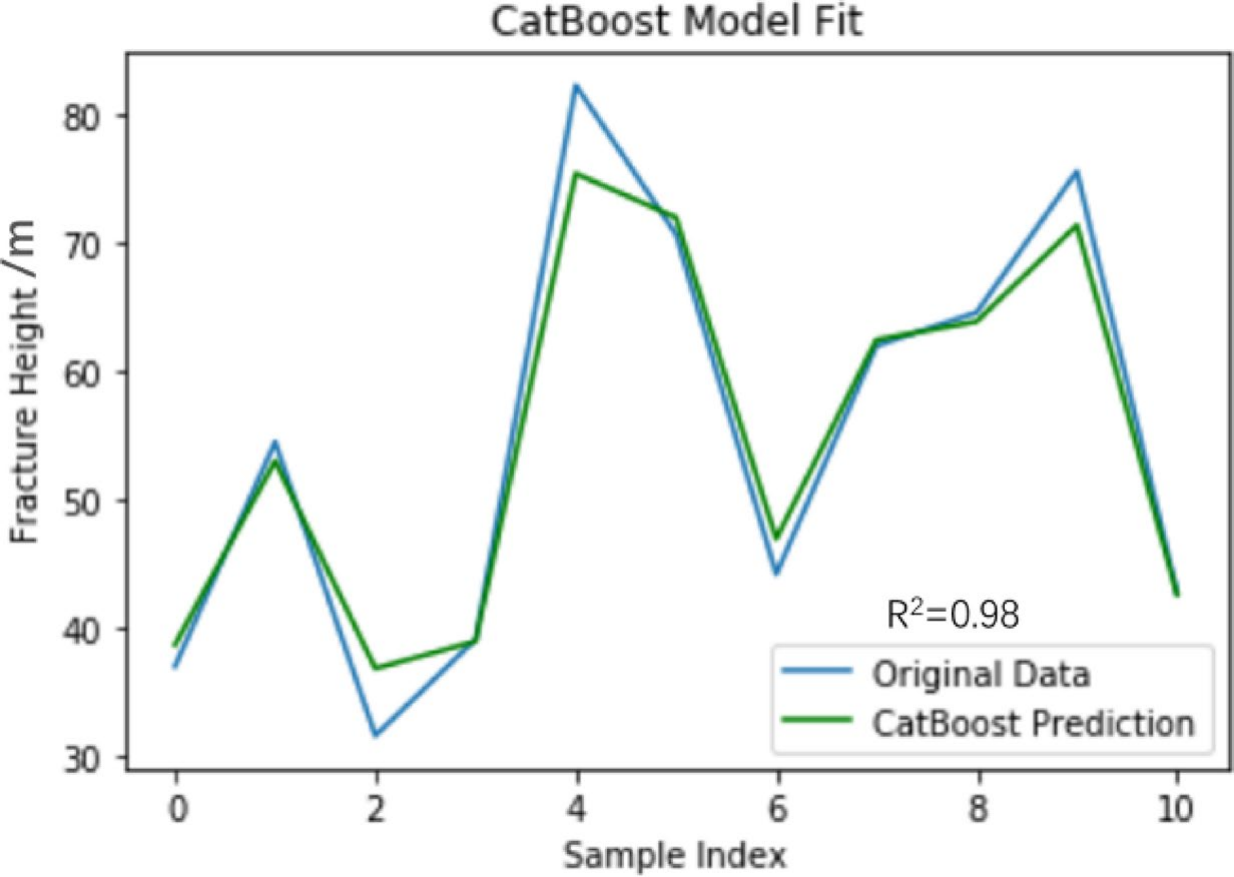
Integrated model fitting diagram.

The integrated model uses CatBoost Regressor, which has a random number seed of 42, a depth of 4, a learning rate of 0.01, and 400 iterations. Substituting into the integrated model, training and testing were conducted using the data in Table 1. Based on the integrated model to predict the height value of the fracture zone, the goodness of fit R^2^, root mean square error (RMSE), MSE, MARE, SI, NSE, and PBIAS were 0.98, 2.08, 4.32, 3.66%, 0.04, 0.98, and − 0.12% respectively. The fitting graph of the prediction results of the integrated model and the true values is shown in Fig. 13. The predicted height of the water-conducting fracture zone in the No. 3 coal seam of Husheng Coal Mine is 50.79 m.The relative error rate compared with the actual fracture zone height is only 3.86%.

To ensure the validity of the test results, cross-validation was conducted using the same model parameters. 20% of the data was used as the test set, and the remaining 80% of the data was used as the training set. Table 5 shows the accuracy of the model trained by cross-validation. Finally, the average prediction accuracy of the test set is 96.67%, which indicates that the integrated model has a relatively good prediction accuracy ability.

**Table 5:** Cross-validation results of the integrated model.

Fold	Accuracy
1	97%
2	95%
3	98%
4	97%
5	96%
Average	96.6%

#### Model uncertainty analysis

To further evaluate the stability and reliability of the constructed ensemble learning model in the height prediction of water-conducting fracture zones, this paper introduces the Bayesian Optimization method to conduct uncertainty analysis on the model. This method is not only used for hyperparameter tuning, but also uses its probabilistic modeling characteristics (Gaussian Process) to evaluate the confidence interval and stability performance of the model’s predicted output under different input combinations.

Bayesian optimization uses the Gaussian process (GP) as the surrogate model. In each round of iteration, the posterior distribution of the objective function is constructed based on the current samples, and the exploration and utilization are balanced through acquisition functions (such as EI, UCB), thereby selecting the optimal parameters. In this paper, RMSE is taken as the objective function to jointly optimize the key hyperparameters of the integrated model (such as max_depth of XGBoost, C of SVR, learning_rate of CatBoost, etc.), and the variance and mean of the model output under each group of parameters are recorded simultaneously.

During the optimization process, by sampling the predicted mean and variance of the Gaussian process, the confidence intervals (95%CI) of the predicted values of the water-conducting fracture zone under different combinations of input features were obtained. Based on the predictions under the optimal model parameters, the prediction fluctuations under multiple samples were statistically analyzed, and the following indicators were obtained:

The predicted mean µ = 50.92 m.

The predicted standard deviation σ = 1.43 m.

Confidence interval CI95%≈[48.12 m, 53.61 m].

This result indicates that the model prediction is overall stable under input disturbances, with small fluctuations, suggesting that the model has strong robustness. Through Bayesian uncertainty analysis, not only the reliability of the model’s prediction results was verified, but also a quantitative basis was provided for the risk definition of the fracture zone height in engineering practice. The provision of confidence intervals can assist in formulating more scientific water prevention and control plans, reduce resource waste caused by excessive safety boundaries, and enhance the interpretability and application value of the model in practical engineering.

## Height prediction of water conduction fracture zone based on FLAC3d

To study the development of the water-conducting fracture zone in the overlaying strata after mining, a suitable prediction method for the height of the water-conducting fracture zone was selected to improve the prediction accuracy. FLAC3d numerical simulation was used to simulate the water-conducting fracture zone of the No. 3 coal seam in Husheng Coal Mine.

Based on the Mohr-Coulomb fault model, the influence of mining activities on the overburden structure is discussed.The unique structural characteristics of the “three zones” in the complex rock mass are revealed by simulating the stress state, displacement characteristics and plastic deformation area of the rock mass. The location of the water-conducting fracture zone near the No. 3 working face is accurately determined, and the height of the fracture zone is predicted. The theoretical study of water-conducting fracture zones is improved by simulating the formation and development of tectonic zones.

### Numerical model establishment

(1) Stress-strain characteristics of rock mass in caving zone.

Because the rock mass in the caving zone has nonlinear compression characteristics, the elastic modulus of loose rock mass changes during compression, improving its supporting ability. The compressive deformation characteristics interact with the supporting attributes of caving rock mass, so the material mechanical properties change dynamically during the compression process, and dynamic assignment is required.

In FLAC3D, the elastic parameters include the volume modulus K and the shear modulus G, and the volume modulus and the shear modulus have the following relationship with the material’s stress-strain.

26 $${\sigma _v}=(K+\frac{{4G}}{2})\varepsilon$$

Assuming Poisson’s ratio v = 0.2,

27 $$G=\frac{3}{4}K$$

The two forms are obtained simultaneously

28 $${\sigma _v}=2K\varepsilon$$

Thus the bulk modulus K and shear modulus G, as well as the vertical stress, can be expressed as a function of the vertical strain $\varepsilon$.

29 $$K=\frac{{4G}}{3}=\frac{{{\sigma _v}}}{{2\varepsilon }}=\frac{{{E_0}\varepsilon }}{{2(1 - \varepsilon /{\varepsilon _m})}}$$

**Fig. 14:**
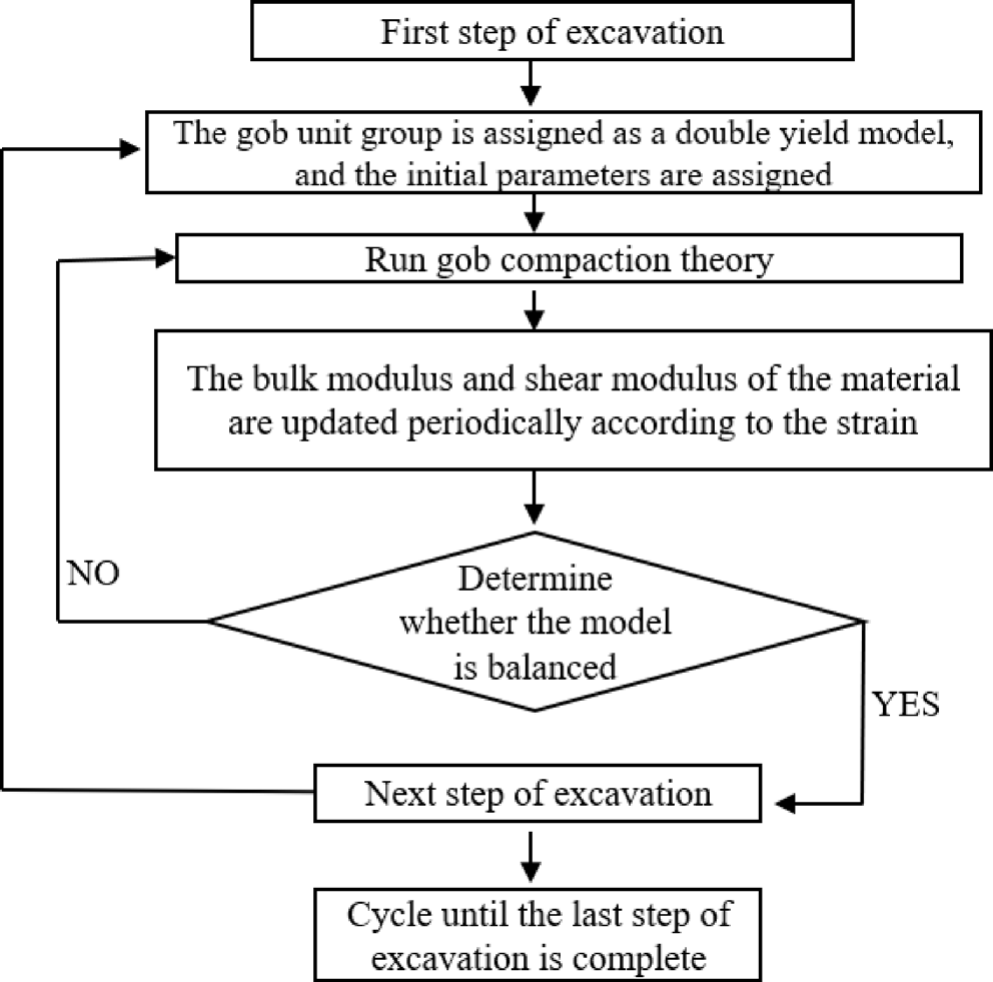
Flow chart of theoretical calculation of goaf compaction.

According to the excavation distance, strain monitoring points are set in the goaf in the model. Fish language programming is used to identify and extract the monitoring point data, which is carried out in a fixed time step. The results are fed back into the above calculation formula of volume modulus and shear modulus, and the material parameters of the goaf are dynamically modified. The theoretical calculation process of goaf re-compaction is shown in Fig. 14.

(2) The boundary conditions of the calculation model are determined.

Based on the geological conditions of the working face studied earlier, the model rock strata were simplified and a calculation model with dimensions of 260 m (x) ×600 m (y) ×140 m (z) was established. The No. 3 coal seam where the 316 working face is located is a shallow-buried deep coal seam with a coal seam dip Angle of 1 to 3°. Therefore, for the convenience of calculation, the dip Angle of the coal seam is set to nearly horizontal and the double yield model is adopted.

Lower boundary conditions: The bottom is fixed support, and the velocity in the x and y directions is 0. Boundary conditions on both sides: Both sides are displacement boundary conditions, and the velocity in the x direction is 0.

To ensure the reliability of the simulation results, finer grids were used in key areas (such as near the water-conducting fracture zone) to provide higher spatial resolution. However, the selection of grid size has a significant impact on the simulation results. Although a smaller grid size can provide a more refined stress-strain distribution, the computational cost also increases significantly. Therefore, in order to ensure the computational efficiency and accuracy of the model, we used larger grid sizes in other areas.

### Analysis of simulation results

#### Study on evolution law of overburden stress in coal seam mining

**Fig. 15:**
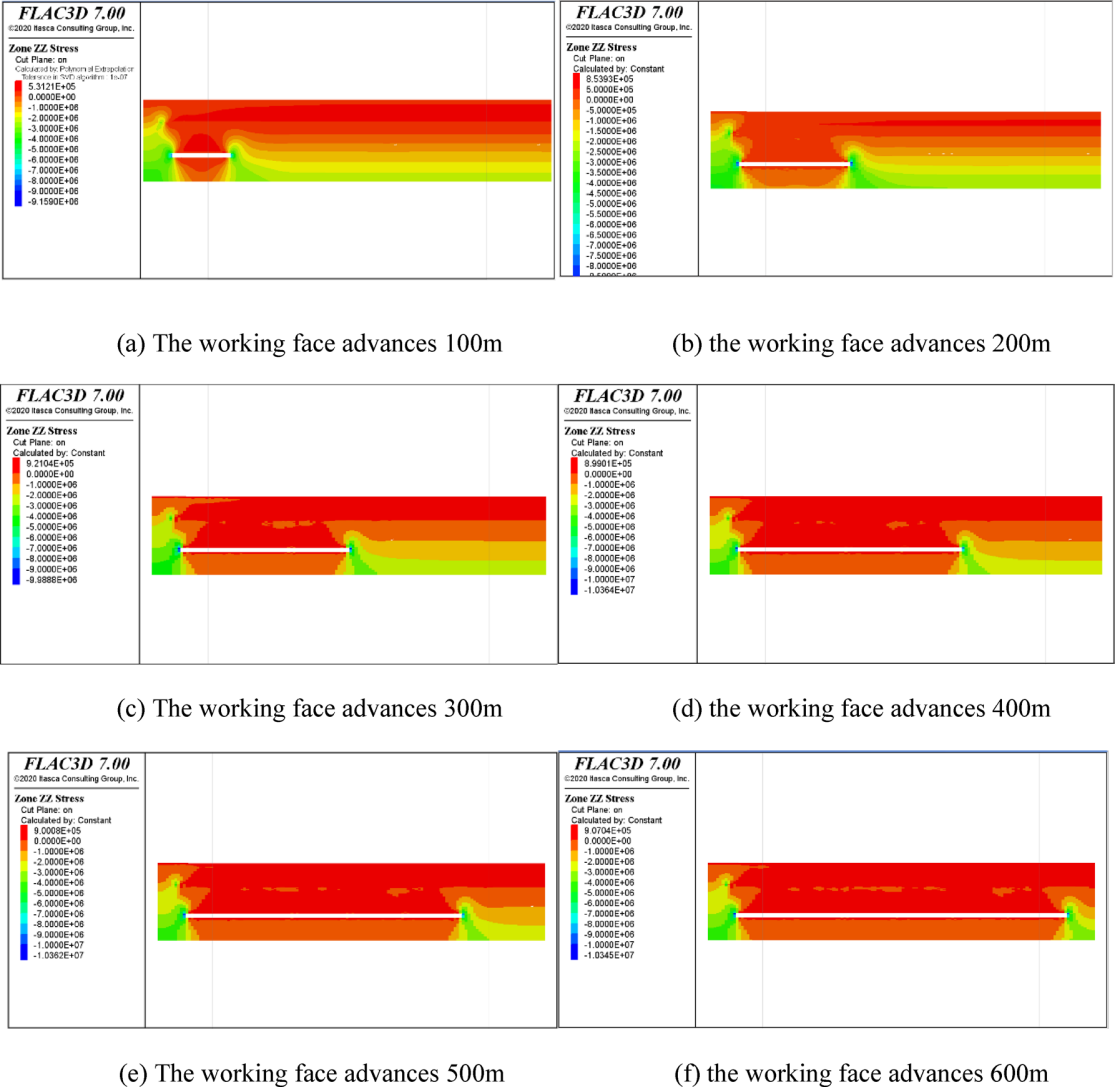
Stress evolution during the advancing process of the working face.

Figure 15 shows the evolution process of vertical stress in the upper rock layer while advancing the working face. During the advancement of the working face, the pressure relief valve of the top rock layer is gradually relieved. When the working face is pushed to 100 m, the pressure relief value of the top rock layer is about 0.07 MPa. The pressure relief valve is about 0.3 MPa, and the pressure relief range is about 55 m. Figure 15 (f) shows two pressure relief zones within the overall pressure relief zone of the roof. The lower zone near the working face is more obvious for pressure relief, which can be regarded as the caving zone, which is 24 m above the roof, and a general pressure relief zone above this range, which can be viewed as the fracture zone, which is 24–55 m above the roof.

#### Study on the evolution law of overburden plastic zone in coal seam mining

**Fig. 16:**
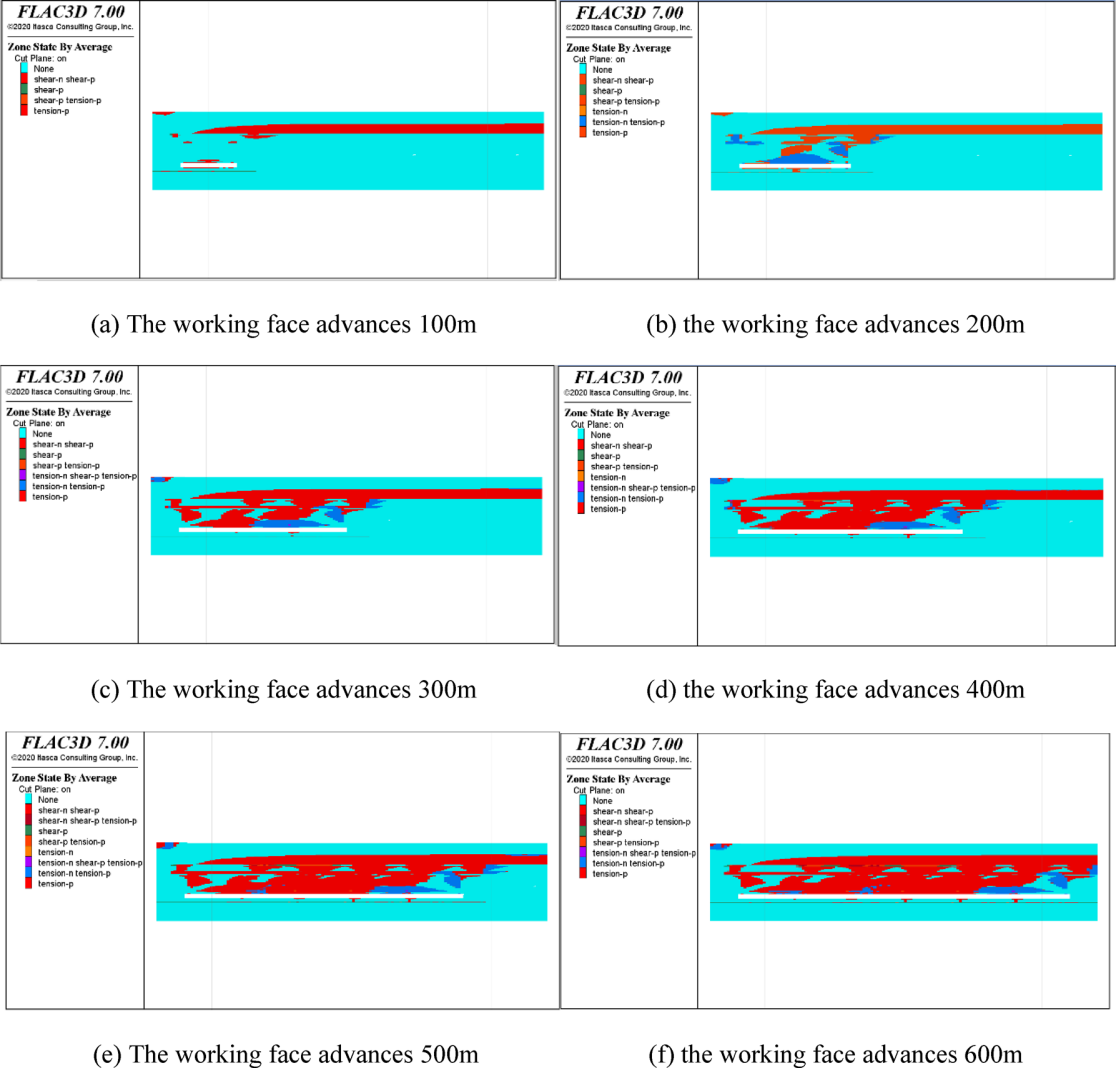
Evolution of the plastic zone during the advancing of the working face.

Figure 16 shows the evolution law of the plastic zone of the roof during the advancing of the working face. With the advancing of the working face, the range of the plastic zone of the roof strata gradually increases, and tensile failure mainly occurs on the top of the working face, while shear failure mainly occurs on both sides. At the end of mining, the tensile failure area of the goaf is connected with the shear failure at the edge of the working face, so the plastic zone near the boundary of the mining range cannot be used as the basis for determining the failure range of “three zones”. The central part of the goaf is selected to determine the range. According to Fig. 16f, the blue area is the area where tensile failure is taking place, and the range of this area is 55 m, so it is judged to be the range of the fracture zone.

#### Study on the evolution law of overburden movement and deformation in coal seam mining

**Fig. 17:**
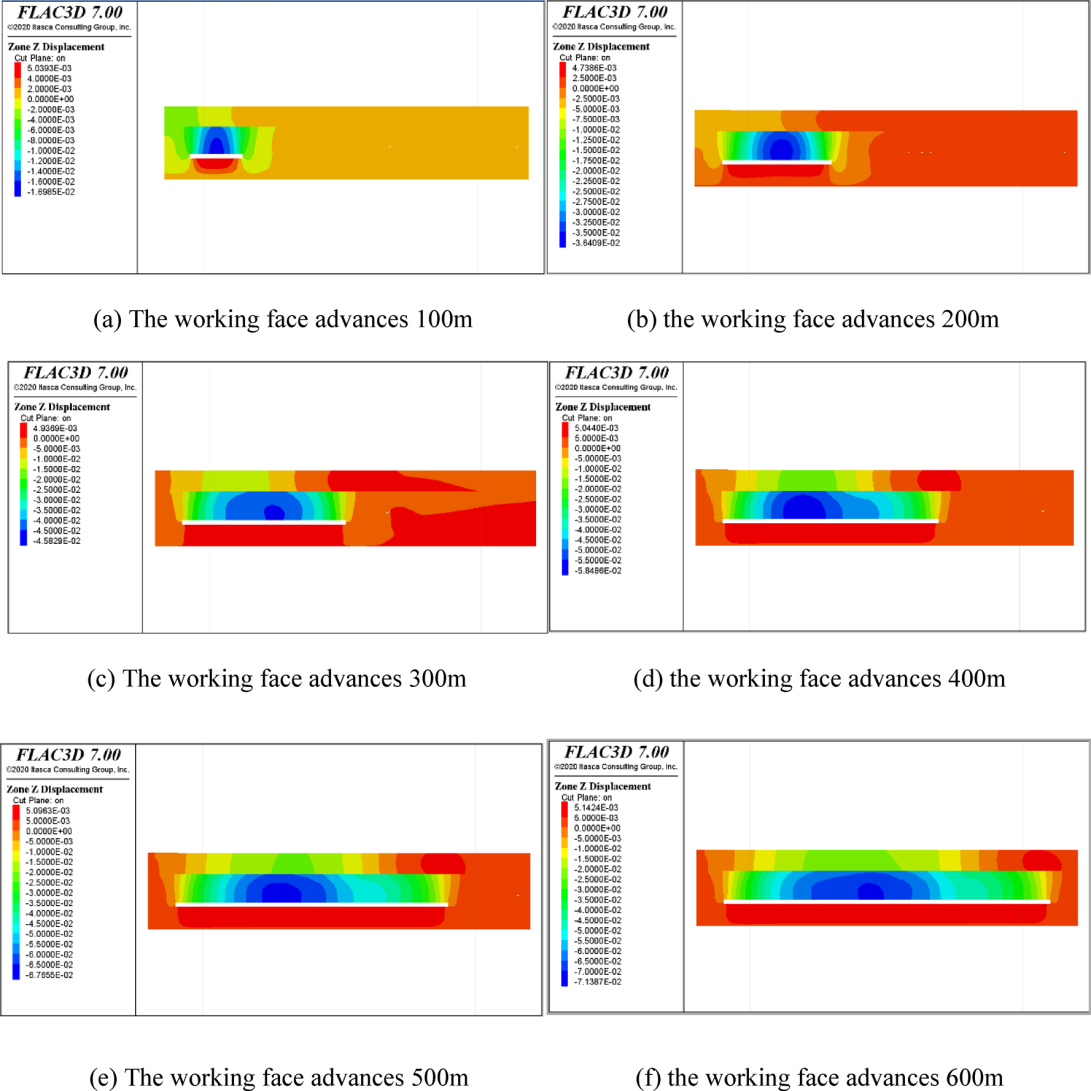
Evolution of strata displacement during the advancing of the working face.

Figure 17 shows the evolution law of displacement and deformation of the roof while advancing the working face. When the working face is advanced to 100 m, the maximum displacement of the roof strata is 0.16 m; when the working face is advanced to 500 m, the maximum displacement of the roof strata is up to 0.6 m—increased to 0.7 m. According to the displacement characteristics of the caving zone and fracture zone, the movement range of the caving zone is the largest before the caving zone and fracture zone are not compacted. Then, the movement range of rock strata in the fracture zone gradually decreases and changes evenly. Therefore, the caving zone and fracture zone range can be determined according to the uniformity of displacement of rock strata before compaction. According to Fig. 17f, the displacement of rock strata within 23 m above the roof is significantly greater than that of rock strata above 24 m ~ 55 m, and the displacement of rock strata above 55 m is small, so it is determined that the range of 24 m above the roof is a caving zone. The range of 24  ~ 55 m is fracture zone.

Finally, according to numerical calculation, when No. 3 coal is mined, the caving zone is 24 m above the roof, the fracture zone is 24 m ~ 55 m above the roof, and the bending subsidence zone is 55 m above the roof.

### Numerical simulation conclusion

Based on the analysis of the evolution law of stress, plastic zone and displacement in the mining process, combined with the load and evolution law of surrounding rock in caving zone and fissure zone, it is concluded that the caving zone is within 24 m above the roof of No. 3 coal seam, the fracture zone is within 24 m ~ 55 m above the roof, and the bending subsidence zone is beyond 55 m.

## Comparison of benchmark test results and on-site verification

### Comparison of benchmark test results

The model results were benchmarked against 10 similar studies, and the performance advantages of the model were determined by two evaluation indicators, namely goodness of fit R2 and root mean square error (RMSE), as shown in Table 6.

**Table 6:** Comparison table of benchmark test Results.

Serial number	Model type	*R* ^2^	RMSE	Main advantages/limitations	Source of literature
1	Convolutional neural network	0.97	6.62	It is more suitable for large-scale data set scenarios with clear spatial structures	Mao et al.^42^
2	Bayesian model	0.96	21.84	In practical application, it is necessary to closely combine with the geological mechanism	Xu et al.^43^
3	PSO-SVR model	0.91	0.53	The application needs to balance data quality, feature engineering and physical mechanism verification	Meng et al. ^44^
4	SVR	0.9122	4.84	Nonlinear patterns in the data can be captured	This article
5	XGBoost	0.9142	4.79	It can handle complex feature interactions	This article
6	This research model	0.98	2.08	Multi-model collaborative optimization	This article
7	Numerical simulation	–	–	The calculation takes a lot of time and is significantly affected by the accuracy of rock mass parameters	This article
8	Multiple linear regression model	0.885	39.21	Poor generalization ability	Wang et al. ^45^
9	SMOGN-BP neural network	0.729	0.487	It is prone to fall into local optimum and has a relatively weak generalization ability	Liu et al. ^46^
10	MPSO-BP neural network	0.901	0.341	Improve the distribution characteristics of the original data set and enhance the predictive performance of the model	Liu et al. ^46^

After comparing the predictive performance of various models for the height of the water-conducting fracture zone, it was found that the model proposed in this study performed the most superior. Specifically, the determination coefficient (R²) of this model reaches 0.98, the highest among all models, and the root mean square error (RMSE) is 2.08, which is also at a relatively low level, demonstrating extremely strong fitting ability and prediction accuracy. In contrast, although models such as convolutional neural networks (R² = 0.97) and Bayesian models (R² = 0.96) also have strong predictive capabilities, their RMSes are 6.62 and 21.84 respectively, and the prediction errors are significantly higher than those of the model in this study. Traditional methods such as multiple linear regression and numerical simulation have obvious deficiencies in terms of accuracy and applicability. Furthermore, although some improved neural network models (such as MPSO-BP) are small in RMSE, their R² values are low and the fitting effect is limited. Overall, through the multi-model collaborative optimization method, this research model not only improves the prediction accuracy but also enhances the robustness and generalization ability of the model. It is superior to the existing mainstream prediction methods and has strong potential for engineering applications.

### Field test

**Fig. 18:**
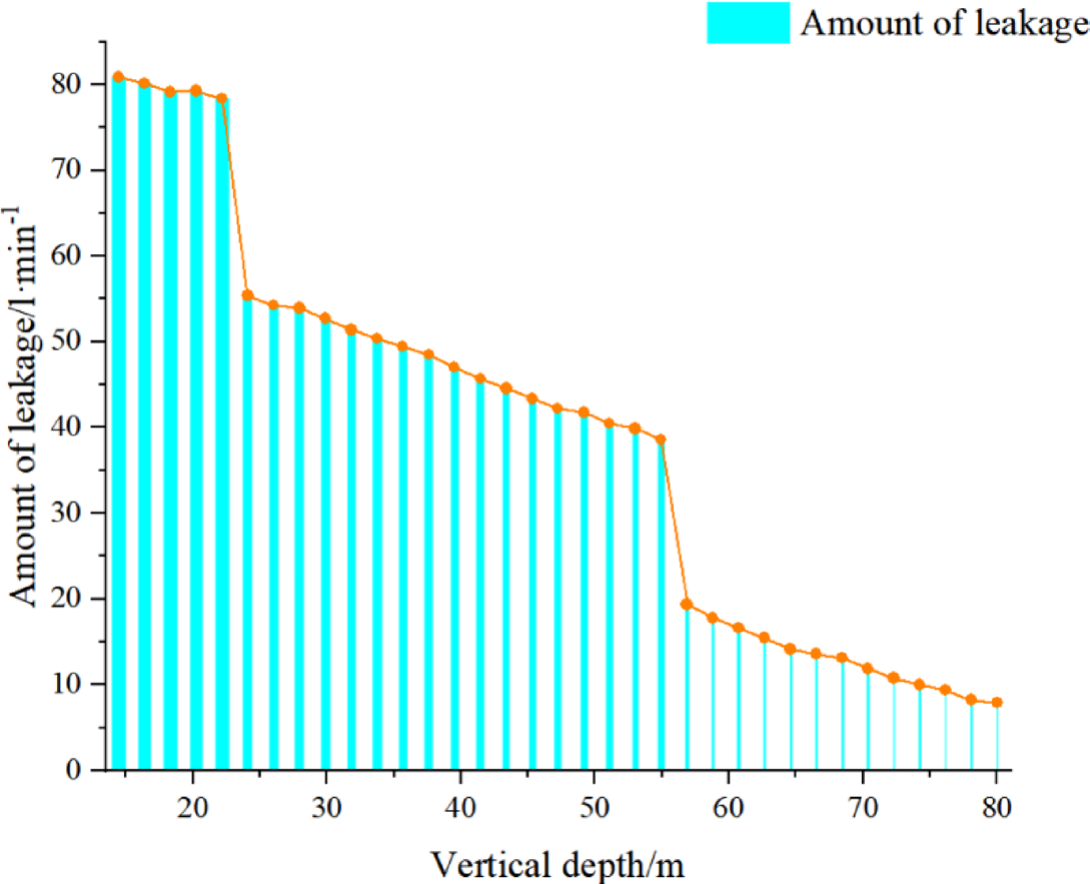
Schematic diagram of leakage and hole depth of No. 2 coal.

Compared with the above prediction results, the integrated model has high accuracy in predicting the height of the water-conducting fracture zone. Therefore, the model indicates the height of the water-conducting fracture zone of the No. 2 coal seam in the study area. In the No. 2 coal seam, the height of the water-conducting fracture zone of the No. 206 working face of the coal seam is also measured according to the segmented injection borehole detection method in the underground overhead hole. The greater the amount of leakage, the height of the fracture zone of the No. 2 coal seam. The relationship between leakage and hole depth is shown in Fig. 18. At the hole depth of 54.37 m, the leakage increases suddenly, reaching 38.58 L/min. It is judged that the height of the fracture zone at this position reaches the maximum. The caving zone height is considered to be 22.18 m, and the results are shown in Table 7.

**Table 7:** Comparison between prediction and measured height of water-conducting fracture zone.

Research area	Mining height /m	Burial depth /m	Face length /m	Lithology proportion factor	Predict the height of water conduction fracture zone /m	Actual water conduction fracture zone height /m	Relative error
No. 2 coal	7.5	82	150	0.445	56.58	54.37	4.06%

The model predicts the height of the water-conducting fracture zone at the 206 working face of the No. 2 coal seam to be 56.58 m. The comparison between the height of the water-conducting fracture zone obtained by field tests and the predicted fracture zone is verified, and it can be concluded that the prediction accuracy of the integrated model is reasonable and reliable. This can provide a practical basis and guidance for calculating the water-conducting fracture zone in this area.

## Conclusion and discussion

### Conclusion

1. This study introduces the stacked ensemble learning method for the first time in the height prediction of water-conducting fracture zones. It integrates SVR and XGBoost as the basic models and takes CatBoost as the meta-model to construct a prediction framework with high accuracy and strong generalization ability, effectively overcoming the problem of insufficient stability of a single model under complex geological conditions.
2. The integrated model outperforms the traditional empirical formula and the single machine learning model in multiple error evaluation indicators, demonstrating a higher degree of fit and lower prediction error, and significantly improving the characterization accuracy of the development height of the actual water-conducting fracture zone.
3. Through comparative analysis with the FLAC3d numerical simulation results, the validity and reliability of the integrated model were verified. Meanwhile, combined with the analysis of the three-band structure, it provided theoretical support for the fracture development mechanism and enhanced the explanatory ability of the model.
4. Through the comparison of benchmark test results and the analysis of relevant studies, it is found that the model in this study, through the multi-model collaborative optimization method, not only improves the prediction accuracy but also enhances the robustness and generalization ability of the model. It is superior to the existing mainstream prediction methods and has strong potential for engineering applications.
5. This research not only enriches the application forms of machine learning in the prediction of coal mine rock layer failure, but also provides a demonstration for the integration of data-driven and mechanism simulation, laying a foundation for the subsequent research on multi-source information collaborative prediction.

### Discussion

1. The success of the integrated model in mine tests has demonstrated its application potential in water hazard prevention and control, providing engineers with economical and effective tools and reducing reliance on complex simulations. However, the model relies on high-quality training data, especially in areas where the geological environment is not adequately represented.
2. Subsequent research will combine the integrated model with real-time monitoring data (such as Internet of Things sensors) to further enhance the dynamic prediction capability; And extend the model to different mining environments to enhance its adaptability and portability.

## Data Availability

All data generated or analyzed in this study are included in the main text.

## Abbreviations

WCFZ: Water-Conducting Fracture Zone – A fractured rock zone formed above mined coal seams, which has the ability to conduct water and poses a risk for mine water inrush
SVR: Support Vector Regression – A supervised learning algorithm based on support vector machines, used for regression tasks
XGBoost: Extreme Gradient Boosting – An efficient and scalable gradient boosting framework that uses tree-based models for regression and classification
CatBoost: Categorical Boosting – A gradient boosting algorithm designed to handle categorical features with high accuracy and speed
RMSE: Root Mean Square Error – A metric used to evaluate the average magnitude of prediction errors in a regression model
R²: Coefficient of Determination – A statistical measure that indicates how well the regression predictions approximate the real data points
FLAC3D: Fast Lagrangian Analysis of Continua in 3 Dimensions – A numerical modeling software used for geotechnical analysis of soil, rock, and structural behavior
ML: Machine Learning – A field of artificial intelligence that uses statistical techniques to give computer systems the ability to learn from data
EL: Ensemble Learning – A machine learning technique that combines predictions from multiple models to improve overall performance
